# Supplementary material for: Incidence of oncogenic HPV infection in women with and without mental illness: A population-based cohort study in Sweden
Source: PLoS Med. 2024 Mar 25;21(3):e1004372. doi: 10.1371/journal.pmed.1004372 (PMC11259452; doi:10.1371/journal.pmed.1004372)
Supplement: S1 Statistical Analysis Plan — (DOCX) [file pmed.1004372.s002.docx]

Analysis_plan_HPVrisk_MD.rtf

Created: 2022-06-07 by Eva Herweijer

Updated: yyyy-mm-dd by First-name Surname

**RISK OF HPV INFECTION IN WOMEN WITH MENTAL DISORDERS**

# BACKGROUND

The first organized cytology-based cervical screening recommendations were issued in 1967. In 2015, the screening recommendations, then dating back from 1998, were updated and screening was no longer solely based on cytology. Women over the age of 30 were recommended to undergo primary HPV-based screening, where HPV-positive women were followed-up by regular cytology. Though the screening recommendation was issued in 2015, primary HPV-based testing was not fully implemented until 2021. More recently, new screening recommendations have been issued by Socialstyrelsen, entirely replacing cytological screening by HPV-based screening, and extending the screening interval. The new recommendation will be effective as of Spring 2022. An overview of the cervical screening recommendation from 1998 and onwards is shown in Table 1.

**Table 1. Overview of screening recommendations by year of implementation**

| **Year of implementation** | **Screening recommendation** |
| --- | --- |
| 1998 | - Ages 23-49: cytology every 3 years - Ages 51-60: cytology every 5 years |
| 2015 | - Ages 23-29: cytology every 3 years - Ages 30-49: HPV test every 3 years. HPV test + cytology at the sampling time closest to age 41 years - 50-64: HPV test every 7 years.   Triage: HPV positive women are followed up with cytology, HPV testing is used as triage for cytology’s with LSIL. |
| 2022 | - HPV testing ages 23-64 years - Ages 23-49: HPV test every 5th year - Ages 50-64: HPV test every 7th year |

# OBJECTIVE AND HYPOTHESES

The objective of the study is to compare the risk of high-risk HPV infection (hereafter referred to as HPV infection) among women with and without mental disorders in a cervical screening cohort

It is hypothesized that the risk of HPV infection is higher among women with mental disorders as compared to women without mental disorders attending cervical screening.

# NOTATION AND ABBREVATIONS

XXX explanation

Q1, Q2,... The different questions in the questionnaire

# STUDY POPULATION

This will be a cohort study including women of ages 30-64 with a negative HPV test result. The study cohort will be limited to the greater Stockholm area representing about 20% of the Swedish population, as (1) primary HPV-based screening has been fully operational as of 2017, and (2) there is additional data available on primary HPV-based screening from the randomized implementation trial that ran during 2012-2016. This randomized implementation of primary HPV-based screening initially targeted women at ages 50-64 years but continued as a full-scale randomized implementation trial targeting all women 30-64 living in the greater Stockholm area from May 2014. The study period will therefore be May 2014 to December 2019. Start of follow-up will be defined by the latest of, a first negative HPV test, or May 1, 2014 if a negative HPV test result was recorded prior to start of the study period. End of follow-up will be the date of positive HPV test result, one screening interval (3 years at ages 30-49 years, 7 years at ages 50-64 years) + 0.5 years after the last negative HPV test result, 65^th^ birthday, moving date outside greater Stockholm region, death, or end of study period, whatever occurred first. Women initially testing HPV positive but subsequently test negative will be eligible for study inclusion (study entry will be the date of first negative test).

## INCLUSION CRITERIA

- Women with a negative HPV test result between May 2014 and 2019, and
- are between ages 30-64 years at the HPV test date, and
- are between ages 30-64 at study entry, and
- are residing in the greater Stockholm area at study entry.

## EXCLUSION CRITERIA

- Women with an inconsistent PNR (REUSED_PNR==1).

# MEASUREMENTS AND VARIABLES

## Outcome

The main outcome of the study will be a high-risk HPV infection regardless of the exact genotype. In Sweden, the Cobas 4800 HPV Test (Roche Molecular Systems, South Branchburg, New Jersey, USA) is used to test for HPV. The Cobas system does partial genotyping and reports the test as HPV16 positive, HPV18 positive and other high-risk HPV positive (HPV types: 31, 33, 35, 39, 45, 51, 52, 56, 58, 59, 66 and 68).

The main study outcome will have the following values:

0 – high-risk HPV negative

1 – high-risk HPV positive

## Exposure covariates

A **diagnosis of a mental health disorder** will be considered the exposure. This will be defined by identification of a clinical diagnosis of a mental health disorder using the national inpatient and outpatient registers.

The clinical diagnoses considered a mental health disorder are shown in **Table 1** below as well as the ICD8/9/10 codes that will be used for the identification as such. Both main and secondary diagnoses will be considered. A patient is considered to be exposed at the date of first diagnosis with the mental health disorder of interest.

**Table 2. Pre-defined mental health disorders and corresponding ICD8/9/10 code**

| Disorder | ICD-8 (1968-1986) | ICD-9 (1987-1996) | ICD-10 (1997-) |
| --- | --- | --- | --- |
| *Any mental disorder* | 291, 295-315 | 291, 292, 295-319 | F10-F99 |
|  |  |  |  |
| *Psychiatric disorders* |  |  |  |
| Substance abuse (not incl. tobacco and alcohol) | 304 | 292, 303, 304, 305X | F10-F19 excl. F10, F17 |
| Alcohol | 291, 303 | 305B, 291 | F10 |
| Tobacco | - | 305A | F17 |
| Psychotic disorders (schizophrenia and non affective psychotic disorders) | 295, 297, 298, 299 | 295, 297, 298 | F20-F29 |
| Depressive disorder | 296.0, 296.2, 298.0, 300.4 | 296B, 296D, 298A, 300E, 311 | F32-F33 |
| Anxiety disorder | 300.0, 300.2 | 300A, 300C | F40-F41 |
| Stress-related disorders | 307 | 308, 309 | F43 |
|  |  |  |  |
| *Neurodevelopmental disorders* |  |  |  |
| Attention-deficit hyperactivity disorder | - | 314 | F90 |
| Autism spectrum disorder | - | 299 | F84 |
| Intellectual disability (mental retardation) | 310-315 | 317-319 | F70-F79 |

Dispensation of **psychiatric medication,** identified from the prescribed drug register, will serve as a proxy for treatment of psychiatric symptoms in primary care. The type of medication and corresponding ATC codes are shown in **Table 3** below. Any dispensation of a pre-defined pharmaceutical prescription will be considered (variables ATC and EDATE).

**Table 3. Pre-defined medication groups that serve as a proxy for mental health disorders and corresponding ATC codes**

| Psychiatric medication | ATC |
| --- | --- |
| *Any psychiatric medication* | N06A*, N05B*, N05C*, N05A* |
|  |  |
| Antidepressant medicine | N06A* |
| Anxiolytics medicine | N05B* |
| Hypnotics and sedatives medicine | N05C* |
| Antipsychotics medicine | N05A* |

Clinical diagnosis of a mental disorder and use of psychiatric medication will be handled as time-varying exposures. A woman will be considered exposed on the first date the diagnosis was given, or first recorded dispensation date of a psychiatric medication.

## Covariates, confounders

**Table 4. Overview of potential study confounders**

| Covariates | Measurement | Source |
| --- | --- | --- |
| Age at index | Index date (YYYYMMDD) - birth date (YYYYMM15) reported in years  To be reported both on continuous scale and categorical scale:   - 23-28 years old - 29-39 years old - 40-49 years old - 50-60 years old | NKCx |
| Country of birth | Country of birth   - Sweden - Other countries | TPR |
| Education level | Highest obtained educational degree at index date (ExamAr, From 2000-2018: SUN2000NIVA_OLD, from 2019: SUN2020NIVA)   - Low - primary education 9 years and below (SUN2000NIVA/SUN2020NIVA IN 1,2) - Medium - 2–3 years of secondary schooling (similar to senior high-school) (SUN2000NIVA/SUN2020NIVA IN 3,4) - High - postsecondary education and above (equivalent to university studies) (SUN2000NIVA/SUN2020NIVA IN 5,6) - Missing/unknown (SUN2000NIVA/SUN2020NIVA IN 9) | LISA/education register |
| Maternal history of CIN3+ | History of histology confirmed CIN3+ of the mother of the study participant prior to study inclusion, confirmed by pathology register.   - Yes - No   Biological and adoptive parents will be identified from the Multi-Generation Register. | NKCx/NCR |
| Screening history | Cervical screening (cytological/HPV) in the last screening round prior to the index date (3 years for women ages 30-49 and 7 years for women ages 50-64) (X_SAMPLE_DATE)   - Unscreened - Screened | NKCx |
| Vaccination status | Time varying exposure. Date of vaccination with dose 1. (Gardasil or cervarix) |  |

# DATA MANAGEMENT

Data are stored at kosmos; database=CERVIX_SOCMOB2. Data is located at the MEB server and can be accessed through VDI.

*** raw data files, program files etc. and documentation files ***

The files in this project are stored in P:\xxxx.

The logbook (LOGBOOK_projectname.doc) can be found in Documents folder.

# STATISTICAL ANALYSES

*Descriptive analyses*

The demographic characteristics at study entry will be described overall, and for patients with and without a mental disorder by means of descriptive statistics. For categorical variables frequency tables (absolute and relative frequencies) will be shown and for continuous variables sample statistics (mean, median, standard deviation, interquartile range) will be reported. Comparisons of proportions will be done by using the Pearson’s X2-test, and for continuous variables, statistical comparisons will be conducted by using an independent sample t-test. For all statistical comparisons, a p-value of <= 0.05 will be considered statistically significant.

*Main analyses*

Risk of HPV infection by mental health status will be assessed by using Cox proportional hazards models estimating hazard ratios (HR) and 95% confidence intervals (CI). Attained age will be the main time scale. Clinical diagnosis of a mental health disorder//use of psychiatric medication will be treated as time-varying exposures. Crude HRs for HPV will be estimated by using univariate Cox regression models for any mental disorder, by type of mental disorder, by use of any psychiatric medication, and by use of groups of psychiatric medication. Similarly, adjusted HRs will be estimated by using multivariate Cox regression models. Covariates that will be considered as part of the multivariate analyses are listed in **Table *4***. Final model selection will be based on inspection of the AIC, and clinical significance. Violation of the proportional hazard assumption will be tested for by inspection of the Schoenfeld residuals and tests of non-zero slopes.

*Sensitivity analyses*

To assess whether the risk of HPV by mental health state would be modified by sociodemographic factors, HRs comparing risk of HPV infection by mental disorder will be estimated stratified by the sociodemographic factors of interest (see **Table *4***). Furthermore, severity of mental disorders and risk of HPV infection will be further investigated by classifying mental disorders as attended in outpatient care (without inpatient care) and attended in inpatient care (with or without outpatient care). Severity of mental disorders and risk of HPV infection will also be investigated by classifying patients with mental disorders by use of psychiatric medication (without inpatient/outpatient care) and attended in inpatient/outpatient care (with or without use of psychiatric medication). These sensitivity analyses will be carried out by using a similar framework as described in the main analyses section, namely Cox proportional hazards models with attained age as the main time-scale and mental disorders, and use of psychiatric medication as time-varying exposure.

To assess whether the study cohort is representative to the greater Stockholm region in terms of prevalence of mental disorders, we will calculate the proportion of person time exposed within the study cohort and a reference cohort. We will sum the person time of women with a mental disorder (exposed person time) and divide this by the sum of person time within the study cohort (exposed and unexposed person time). The reference cohort will include all women that were between 30 and 65 and were living in the greater Stockholm region at study entry. The start of follow-up will be May 1, 2014, 30^th^ birthday whatever comes last. The end of follow-up will be 65^th^ birthday, moving date outside Stockholm region, death, or end of study period, whatever occurred first. Two-sided 95% Exact confidence intervals for proportions will be calculated.

# STAFF LIST

*****, PI, first author

*****, co-I

*****, Ph D student

*****, data manager

*****, biostatistician

*****, ****other

**APPENDIX 1 – Meeting Minutes**

**APPENDIX 2 – Selected Results**
