# Supplementary figures and images for: Incidence of oncogenic HPV infection in women with and without mental illness: A population-based cohort study in Sweden
Source: PLoS Med. 2024 Mar 25;21(3):e1004372. doi: 10.1371/journal.pmed.1004372 (PMC11259452; doi:10.1371/journal.pmed.1004372)

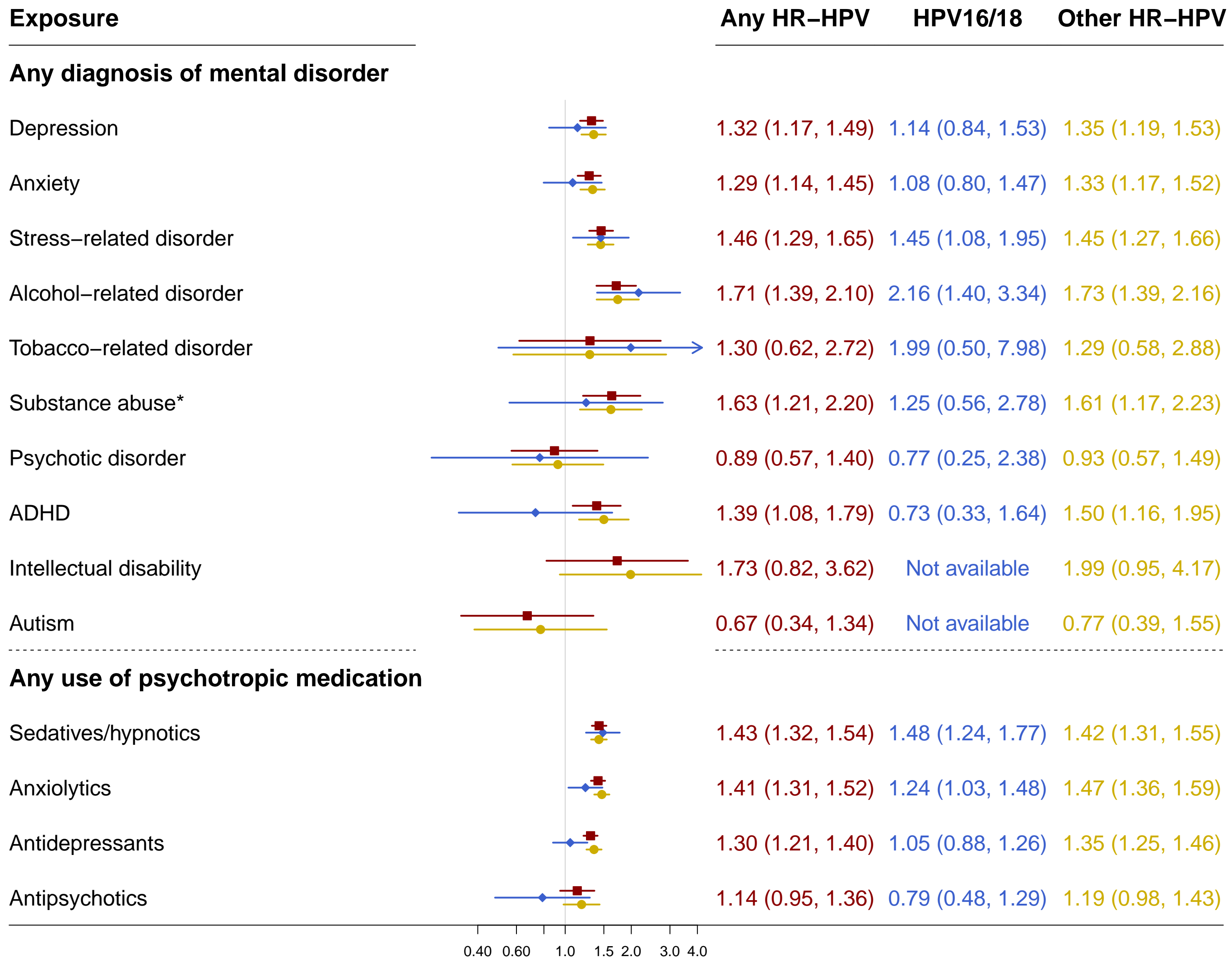

Supplement: S1 Fig — *Tobacco- and alcohol-related disorders are excluded. ADHD, attention-deficit hyperactivity disorder; CI, confidence interval; HPV, human papillomavirus; HR, hazard ratio. HR-HPV, high-risk HPV, including 14 types: 16, 18, 31, 33, 35, 39, 45, 51, 52, 56, 58, 59, 66, and 68. (PDF) [file pmed.1004372.s003.pdf]
