# Supplementary material for: Incidence of oncogenic HPV infection in women with and without mental illness: A population-based cohort study in Sweden
Source: PLoS Med. 2024 Mar 25;21(3):e1004372. doi: 10.1371/journal.pmed.1004372 (PMC11259452; doi:10.1371/journal.pmed.1004372)
Supplement: S1 Table — (DOCX) [file pmed.1004372.s004.docx]

**S1 Table.** **International Classification of Diseases (ICD) codes for pre-defined mental disorders**

| **Category** | **ICD-8 (1968-1986)** | **ICD-9 (1987-1996)** | **ICD-10 (1997-)** |
| --- | --- | --- | --- |
| **Mental disorder** | All of the below | All of the below | All of the below |
| **Psychiatric disorders** |  |  |  |
| Depression | 296.0, 296.2, 298.0, 300.4 | 296B, 296D, 298A, 300E, 311 | F32-F33 |
| Anxiety | 300.0, 300.2 | 300A, 300C | F40-F41 |
| Stress-related disorder | 307 | 308, 309 | F43 |
| Alcohol-related disorder | 291, 303 | 305B, 291 | F10 |
| Tobacco-related disorder | - | 305A | F17 |
| Substance abuse, excl. tobacco- and alcohol-related disorders | 304 | 292, 303-305 except (305A, 305B) | F10-F19 except (F10, F17) |
| Psychotic disorder^1^ | 295, 297, 298, 299 | 295, 297, 298 | F20-F29 |
| **Neurodevelopmental disorders** |  |  |  |
| Attention-deficit hyperactivity disorder (ADHD) | - | 314 | F90 |
| Autism | - | 299 | F84 |
| Intellectual disability | 310-315 | 317-319 | F70-F79 |

^1^ Psychotic disorder includes schizophrenia and non-affective psychotic disorders.
