# Supplementary material for: Incidence of oncogenic HPV infection in women with and without mental illness: A population-based cohort study in Sweden
Source: PLoS Med. 2024 Mar 25;21(3):e1004372. doi: 10.1371/journal.pmed.1004372 (PMC11259452; doi:10.1371/journal.pmed.1004372)
Supplement: S2 Table — (DOCX) [file pmed.1004372.s005.docx]

**S2 Table. ATC codes for pre-defined psychotropic medications**

| Psychotropic medication | ATC |
| --- | --- |
| *Any psychotropic medication* | N06A*, N05B*, N05C*, N05A* |
| Antidepressants | N06A* |
| Anxiolytics | N05B* |
| Hypnotics and sedatives | N05C* |
| Antipsychotics | N05A* |
