# Supplementary material for: Incidence of oncogenic HPV infection in women with and without mental illness: A population-based cohort study in Sweden
Source: PLoS Med. 2024 Mar 25;21(3):e1004372. doi: 10.1371/journal.pmed.1004372 (PMC11259452; doi:10.1371/journal.pmed.1004372)
Supplement: S3 Table — (DOCX) [file pmed.1004372.s006.docx]

**S3 Table. Number of person-years by different characteristics among women with or without diagnosis of mental disorder or filled prescription of psychotropic medication**

| **Characteristics** | **Diagnosis of mental disorder** | | **Use of psychotropic medication** | |
| --- | --- | --- | --- | --- |
|  | **No** | **Any** | **No** | **Any** |
| **Person-years (PYs)** | 668,494 | 110648 | 449011 | 330130 |
|  |  |  |  |  |
| **Age at follow-up, PYs (%)** |  |  |  |  |
| <40 | 187,438 (28·04) | 34,869 (31·51) | 144,423 (32·16) | 77,884 (23·59) |
| 40-49 | 208,168 (31·14) | 34,702 (31·36) | 142,588 (31·76) | 100,283 (30·38) |
| 50-65 | 272,887 (40·82) | 41,076 (37·12) | 162,000 (36·08) | 151,964 (46·03) |
|  |  |  |  |  |
| **Country of birth, PYs (%)** |  |  |  |  |
| Nordic countries | 463,127 (69·28) | 80,454 (72·71) | 306,797 (68·33) | 236,783 (71·72) |
| Other | 205,367 (30·72) | 30,194 (27·29) | 142,214 (31·67) | 93,347 (28·28) |
|  |  |  |  |  |
| **Educational level at cohort entry, PYs (%)** |  |  |  |  |
| Low | 52,799 (7·90) | 12,027 (10·87) | 32,669 (7·28) | 32,157 (9·74) |
| Medium | 218,931 (32·75) | 40,208 (36·34) | 139,912 (31·16) | 119,227 (36·12) |
| High | 387,752 (58·00) | 57,909 (52·34) | 268,536 (59·81) | 177,125 (53·65) |
| Unclassified | 9,012 (1·35) | 503 (0·45) | 7,895 (1·76) | 1,621 (0·49) |
|  |  |  |  |  |
| **HPV vaccination at follow-up, PYs (%)** |  |  |  |  |
| Unvaccinated | 660,637 (98·82) | 108,788 (98·32) | 443,548 (98·78) | 327,260 (98·71) |
| Vaccinated | 7,857 (1·18) | 1,860 (1·68) | 5,463 (1·22) | 4,263 (1·29) |
|  |  |  |  |  |
| **Maternal history of CIN3+ at follow-up, PYs (%)** |  |  |  |  |
| No | 640,329 (95·79) | 104,892 (94·80) | 430,726 (95·93) | 325,877 (98·71) |
| Yes | 28,165 (4·21) | 5,755 (5·20) | 18,286 (4·07) | 4,253 (1·29) |

Abbreviation: CIN3+, cervical intraepithelial neoplasia grade 3 or worse

The following variables are treated as time-varying: mental illness, filled prescription of psychotropic medication, age, HPV vaccination, maternal history of CIN3+**.**
