# Supplementary material for: Incidence of oncogenic HPV infection in women with and without mental illness: A population-based cohort study in Sweden
Source: PLoS Med. 2024 Mar 25;21(3):e1004372. doi: 10.1371/journal.pmed.1004372 (PMC11259452; doi:10.1371/journal.pmed.1004372)
Supplement: S4 Table — CI, confidence interval; HPV, human papillomavirus; HR, hazard ratio. (DOCX) [file pmed.1004372.s007.docx]

**S4 Table. Adjusted hazard ratios (HRs) with 95% confidence intervals (CIs) of high-risk HPV infection in relation to diagnosis of mental disorder or use of psychotropic medication, by attained age**

| Groups | **Any high risk-HPV**^1^ | **HPV16/18** | **Other high risk-HPV** |
| --- | --- | --- | --- |
|  | HR (95%CI)^2^ | HR (95%CI)^2^ | HR (95%CI)^2^ |
| Any diagnosis of mental disorder | | | |
| 30-39 years | 1·28 (1·12-1·47) | 1·33 (0·96-1·83) | 1·31 (1·13-1·52) |
| 40-49 years | 1·38 (1·19-1·59) | 1·02 (0·69-1·49) | 1·44 (1·24-1·69) |
| 50-64 years | 1·43 (1·19-1·71) | 1·57 (1·06-2·35) | 1·39 (1·14-1·70) |
| *P_interaction_^3^* | 0·603 | 0·282 | 0·663 |
| Any diagnosis of psychiatric disorder | | | |
| 30-39 years | 1·28 (1·11-1·47) | 1·37 (0·99-1·89) | 1·30 (1·12-1·51) |
| 40-49 years | 1·38 (1·19-1·60) | 1·04 (0·71-1·53) | 1·45 (1·23-1·69) |
| 50-64 years | 1·43 (1·19-1·71) | 1·60 (1·07-2·38) | 1·39 (1·14-1·69) |
| *P_interaction_^3^* | 0·594 | 0·293 | 0·633 |
| Any diagnosis of neurodevelopmental disorder | | | |
| 30-39 years | 1·30 (0·94-1·80) | 0·56 (0·18-1·77) | 1·42 (1·02-1·98) |
| 40-49 years | 1·25 (0·84-1·88) | 0·84 (0·27-2·64) | 1·34 (0·88-2·05) |
| 50-64 years | 1·07 (0·53-2·14) | - | 1·24 (0·62-2·50) |
| *P_interaction_^3^* | 0·873 | 0·149 | 0·938 |
| Any use of psychotropic medication | | | |
| 30-39 years | 1·42 (1·27-1·58) | 1·47 (1·13-1·91) | 1·46 (1·30-1·64) |
| 40-49 years | 1·46 (1·30-1·64) | 1·19 (0·91-1·56) | 1·50 (1·33-1·70) |
| 50-64 years | 1·43 (1·24-1·64) | 1·15 (0·84-1·58) | 1·47 (1·26-1·71) |
| *P_interaction_^3^* | 0·924 | 0·404 | 0·938 |

Abbreviations: high risk-HPV, high-risk human papillomavirus; HR, hazard ratio; CI, 95% confidence interval

^1^ high-risk HPV includes 14 types: 16, 18, 31, 33, 35, 39, 45, 51, 52, 56, 58, 59, 66, and 68.

^2^ Adjusted for age, country of birth, educational level, HPV vaccination status, and maternal history of CIN3+

^3^ Likelihood ratio test was used to test for interaction between mental health status and age.
