# Supplementary material for: Incidence of oncogenic HPV infection in women with and without mental illness: A population-based cohort study in Sweden
Source: PLoS Med. 2024 Mar 25;21(3):e1004372. doi: 10.1371/journal.pmed.1004372 (PMC11259452; doi:10.1371/journal.pmed.1004372)
Supplement: S5 Table — CI, confidence interval; HPV, human papillomavirus; HR, hazard ratio; IR, incidence rate. (DOCX) [file pmed.1004372.s008.docx]

**S5 Table. Incidence rates (IRs) and adjusted hazard ratios (HRs) with 95% confidence intervals (CIs) of high-risk HPV infection by severity of mental illness**

|  |  | **Any high risk-HPV**^1^ | | **HPV16/18** | | **Other high risk-HPV** | |
| --- | --- | --- | --- | --- | --- | --- | --- |
| Group | *Person-years* | *No. of infection* | *adjusted HR (95%CI)* ^2^ | *No. of infection* | *adjusted HR (95%CI)* ^2^ | *No. of infection* | *adjusted HR (95%CI)* ^2^ |
| **Any diagnosis of mental disorder** | | | | | | | |
| No | 436,011 | 1,557 | Reference | 300 | Reference | 1,332 | Reference |
| Only primary care ^3^ | 232,483 | 1,087 | 1·43 (1·33-1·55) | 189 | 1·28 (1·07-1·54) | 949 | 1·47 (1·35-1·60) |
| Secondary care ^4^ | 110,648 | 619 | 1·54 (1·40-1·69) | 108 | 1·39 (1·11-1·73) | 545 | 1·59 (1·44-1·75) |

^1^ High-risk HPV includes 14 types: 16, 18, 31, 33, 35, 39, 45, 51, 52, 56, 58, 59, 66, and 68

^2^ Adjusted for age, country of birth, educational level, HPV vaccination status, and maternal history of CIN3+

^3^ Only dispensed with psychotropic medication but not attended by a specialist

^4^ Secondary care with or without primary care, reflected by clinical diagnosis by a specialist
