# Supplementary material for: Incidence of oncogenic HPV infection in women with and without mental illness: A population-based cohort study in Sweden
Source: PLoS Med. 2024 Mar 25;21(3):e1004372. doi: 10.1371/journal.pmed.1004372 (PMC11259452; doi:10.1371/journal.pmed.1004372)
Supplement: S6 Table — CI, confidence interval; HPV, human papillomavirus; HR, hazard ratio. (DOCX) [file pmed.1004372.s009.docx]

**S6 Table. Adjusted hazard ratios (HRs) with 95% confidence intervals (CIs) of high-risk HPV infection in relation to diagnosis of mental disorder or use of psychotropic medication, using 12-month grace period to define loss to follow-up**

|  |  | **Any high risk-HPV**^1^ | | | **HPV16/18** | | | **Other high risk-HPV** | | |
| --- | --- | --- | --- | --- | --- | --- | --- | --- | --- | --- |
| Groups | Person-years | No. of infection | Adjusted HR (95%CI)^2^ | P-value | No. of infection | Adjusted HR (95%CI)^2^ | P-value | No. of infection | Adjusted HR (95%CI)^2^ | P-value |
| **Any diagnosis of mental disorder** | | | | | | | | | | |
| No | 682,113 | 2868 | Reference |  | 529 | Reference |  | 2,281 | Reference |  |
| Yes | 113,498 | 673 | 1.34 (1.23-1.46) | p<0.001 | 116 | 1.25 (1.02-1.53) | 0.025 | 545 | 1.37 (1.26-1.50) | p<0.001 |
| **Any diagnosis of psychiatric disorder** | | | | | | | | | | |
| No | 684424 | 2883 | Reference |  | 529 | Reference |  | 2,488 | Reference |  |
| Yes | 111188 | 658 | 1.34 (1.23-1.46) | p<0.001 | 116 | 1.29 (1.05-1.57) | 0.013 | 580 | 1.37 (1.25-1.50) | p<0.001 |
| **Any diagnosis of neurodevelopmental disorder** | | | | | | | | | | |
| No | 783,873 | 3467 | Reference |  | 639 | Reference |  | 2,998 | Reference |  |
| Yes | 11,738 | 74 | 1.20 (0.95-1.51) | 0.123 | 6 | 0.53 (0.23-1.18) | 0.118 | 70 | 1.31 (1.03-1.67) | 0.026 |
| **Any use of psychotropic medication** | | | | | | | | | | |
| No | 458,163 | 1783 | Reference |  | 337 | Reference |  | 1,528 | Reference |  |
| Yes | 337,449 | 1758 | 1.43 (1.34-1.53) | p<0.001 | 308 | 1.32 (1.13-1.54) | p<0.001 | 1,540 | 1.47 (1.37-1.58) | p<0.001 |

^1^ High-risk HPV includes 14 types: 16, 18, 31, 33, 35, 39, 45, 51, 52, 56, 58, 59, 66, and 68

^2^ Adjusted for age, country of birth, educational level, HPV vaccination status, and maternal history of CIN3+
