# Supplementary material for: Incidence of oncogenic HPV infection in women with and without mental illness: A population-based cohort study in Sweden
Source: PLoS Med. 2024 Mar 25;21(3):e1004372. doi: 10.1371/journal.pmed.1004372 (PMC11259452; doi:10.1371/journal.pmed.1004372)
Supplement: Attachment — Submitted filename: Response_to_reviewers.docx [file pmed.1004372.s010.docx]

Manuscript reference number: PMEDICINE-D-23-02359R1

Dear Dr. Janin,

Thank you for the opportunity to submit a revised version of our manuscript "Incidence of oncogenic HPV infection in women with and without mental illness: a population-based cohort study in Sweden" (PMEDICINE-D-23-02359R1) to PLOS Medicine.

We appreciate greatly the constructive comments from the editors and reviewers and have now addressed all comments point-by-point and accordingly made changes to the manuscript. We believe that these revisions have further improved our manuscript and hope that you will find this revised version acceptable for publication.

Yours sincerely,

Kejia Hu, MD PhD

Institute of Environmental Medicine

Karolinska Institutet

Nobels väg 13 | 171 77 Stockholm, Sweden
+46 76 081 07 52
[kejia.hu@ki.se](mailto:kejia.hu@ki.se)

-----------------------------------------------------------

Comments from the Executive editor: Please outline in detail whether and how much this population overlaps with the population studied in the prior Lancet Pub Health paper. It follows that these patients might have been a sub-set of those in the prior paper, and this will be important to acknowledge/discuss. The points made by the statistical reviewer are also very important.

Response: Thanks for the great comment. The Lancet Pub Health paper included 4,112,598 women born during 1940-1995 who ever resided in Sweden during 1968-2018, while in this study we included 337,116 women who were at age 30-64, ever lived in Stockholm, and had a negative HPV test, during 08/2014-12/2019. As the women included in this study were born during 1950-1989, they are indeed a subset of the study population of the previous paper. We have now highlighted this in the Discussion section. We have also carefully addressed all the points made by the statistical reviewer.

*“This is in line with our previous study [3] including a population of 4 million women, showing an increased risk of precancerous cervical lesions among both groups of women.”* (Discussion section, page 12, lines 268-270 in the clean version of the manuscript)

*“Importantly, the 337,116 women included in the present study are a subset of the 4 million women included in our previous study [3] and the findings corroborated with each other.”* (Discussion section, page 14, lines 314-316)

Requests from the editors:

GENERAL:

Please provide 95% CIs and p values for all results were appropriate (including the abstract), check and amend throughout. We suggest reporting statistical information in the following format: ‘x’; (95% CI [‘y’,’ z’] p value) For p values, please report as p<0.001 and where higher as ‘p=0.002’. Please add the statistical method used to your method section.

Response: We have now amended the statistical information according to the required format and provided p values wherever needed. We have further clarified the statistical method in the Statistical Analysis section.

*“**To ensure generalizability of our findings, we first compared the prevalence of mental illness in the study cohort (i.e., women at screening age who tested negative for high-risk HPV at cohort entry) to the prevalence in the alternative female population at screening age in Stockholm (i.e., entire Stockholm female population after excluding our study cohort). We calculated prevalence through dividing exposed proportion of follow-up time by the total follow-time during the study period of both the study cohort and the alternative female population in Stockholm.*

*Within the study cohort, participant characteristics were summarized by the distribution of person-years among women with or without mental illness, using time-varying exposure and other time-varying covariables.* *We fitted multivariable Cox proportional hazards model to estimate the relative infection rate of high-risk HPV in relation to diagnosis of mental disorder or use of psychotropic medication, as hazard ratios (HR) and 95% confidence intervals (CI). We reported p values of the coefficient using likelihood ratio test. We also fitted separate Cox models for HPV-16/18 and other high-risk HPV infections, as positivity of different types of HPV might be reported in one HPV test. We employed attained age as the underlying time scale for our analysis. No major violation of the proportional hazard assumption was found after plotting the Schoenfeld residuals over time.* *Models were implicitly adjusted for age (as age was used as the underlying time scale), and further adjusted for country of birth, educational level, HPV vaccination status, and maternal history of CIN3+. Finally, to examine potentially differential attendance in the subsequent screening between women with or without mental illness, we defined women who ended the follow-up at one screening interval plus six months as loss to follow-up (i.e., missing a subsequent screening) and estimated HR of loss to follow-up in relation to mental illness, using Cox models with the same adjustment.*

*To examine potential effect modification by age, we fitted interaction models by adding an interaction term between age (30-39, 40-49, and 50-64 years) and mental illness. We tested for the statistical significance of the interaction by log-likelihood ratio test. To assess the role of severity of mental illness on HPV infection rate, we performed a sensitivity analysis by analyzing separately women with a specialist diagnosis of mental disorder (regardless of medication use) and women with only filled prescription of psychotropic medications (i.e., without a specialist diagnosis).* *We further performed a sensitivity analysis by censoring at one screening interval plus 12, instead of six, months since the last negative test for high-risk HPV, to allow more time for delay in attending the next screening. We performed an additional sensitivity analysis by treating data as interval censored, i.e., the event could happen any time between the last negative test and the first positive test, or any time after the last negative test. We fitted Cox models* *to estimate the HRs and estimated the standard error using bootstrapping.”* (Methods section, pages 8-10, lines 160-194)

For in-text reference, citations are placed within square parentheses and should precede punctuation. Please amend throughout.

Response: We have amended the format of citations throughout the manuscript.

STUDY DESIGN:

Please ensure that the study is reported according to the STROBE guideline, and include the completed STROBE checklist as Supporting Information. Please add the following statement, or similar, to the Methods: ""This study is reported as per the Strengthening the Reporting of Observational Studies in Epidemiology (STROBE) guideline (S1 Checklist).""

The STROBE guideline can be found here: <https://eur01.safelinks.protection.outlook.com/?url=http%3A%2F%2Fwww.equator-network.org%2Freporting-guidelines%2Fstrobe%2F&data=05%7C01%7Ckejia.hu%40ki.se%7Cfd479f3e66c048e7fa8208dbcbcfa22d%7Cbff7eef1cf4b4f32be3da1dda043c05d%7C0%7C0%7C638327864865895018%7CUnknown%7CTWFpbGZsb3d8eyJWIjoiMC4wLjAwMDAiLCJQIjoiV2luMzIiLCJBTiI6Ik1haWwiLCJXVCI6Mn0%3D%7C3000%7C%7C%7C&sdata=UhBoyCNZuk2vQRg4n7X2cY71yjeDOk%2F9JSqXfRN4904%3D&reserved=0>

When completing the checklist, please use section and paragraph numbers, rather than page numbers."

Response: We have stated that we followed STROBE guidelines and have included the checklist as Supporting Information. Section and paragraph numbers are used in the checklist. We have also added the following statement in the manuscript:

*“This study is reported as per the Strengthening the Reporting of Observational Studies in Epidemiology (STROBE) guideline (S2 Checklist).”* (Methods section, page 10, lines 201-202)

"Did your study have a prospective protocol or analysis plan? Please state this (either way) early in the Methods section.

a) If a prospective analysis plan (from your funding proposal, IRB or other ethics committee submission, study protocol, or other planning document written before analyzing the data) was used in designing the study, please include the relevant prospectively written document with your revised manuscript as a Supporting Information file to be published alongside your study, and cite it in the Methods section. A legend for this file should be included at the end of your manuscript.

b) If no such document exists, please make sure that the Methods section transparently describes when analyses were planned, and when/why any data-driven changes to analyses took place.

c) In either case, changes in the analysis-- including those made in response to peer review comments-- should be identified as such in the Methods section of the paper, with rationale."

For all observational studies, in the manuscript text, please indicate: (1) the specific hypotheses you intended to test, (2) the analytical methods by which you planned to test them, (3) the analyses you actually performed, and (4) when reported analyses differ from those that were planned, transparent explanations for differences that affect the reliability of the study's results. If a reported analysis was performed based on an interesting but unanticipated pattern in the data, please be clear that the analysis was data-driven.

Response: We have uploaded a pre-specified analysis plan as Supporting Information and added the required information in the manuscript.

*“A statistical analysis plan written before analyzing the data can be found in Supporting Information (S2 Analysis Plan). All analyses were performed as planned.”* (Methods section, page 10, lines 196-197)

DATA AVAILABILITY:

The Data Availability Statement (DAS) requires revision. Please include an appropriate contact as a study author cannot be the contact person for the data.

Response: We have now amended the statement.

*“The raw datasets used in the present study are unavailable for sharing because of privacy policies and regulations in Sweden. Please contact Statistics Sweden (www.scb.se/en) and The Swedish National Board of Health and Welfare (*[*www.socialstyrelsen.se/en*](http://www.socialstyrelsen.se/en)*) for potential data access. Updated contact information to access the data can be found through the websites.”* (Metadata in submission form)

ABSTRACT:

Abstract Methods and Findings:

Please include length of follow up

Response: We have now added information on length of follow-up.

*“3,263 women were tested positive for high-risk HPV* *during follow-up (median: 2.21 years; range:* *0-5.42 years).”* (Abstract, page 2, lines 17-18)

Please quantify the main results (with 95% CIs and p values - in line with the comment made above).

Response: We have now quantified the main results as required in the Abstract and throughout the manuscript.

Please clarify when you write ‘risk’ if you are referring to absolute or relative risk

Response: We have now clarified this in the Abstract and throughout the manuscript.

*“We aimed to examine whether women with mental illness had an increased infection rate of HPV compared to women without mental illness.”* (Abstract, page 2, lines 5-6)

*“The absolute infection rate of HPV was higher among women with a specialist diagnosis of mental disorder…”* (Abstract, page 2, lines 18-19)

*“The increment in absolute infection rate was noted for depression,…”* (Abstract, pages 2-3, lines 20-23)

*“We fitted multivariable Cox proportional hazards model to estimate the relative infection rate of high-risk HPV …”* (Methods section, page 9, lines 169-171)

Result: given the study design, it would be beneficial to use associational language for the outcome.

Response: We have now modified it accordingly.

*“Mental illness is associated with an increased infection rate of high-risk HPV in women.”* (Abstract, page 3, line 30)

AUTHORS SUMMARY:

At this stage, we ask that you include a short, non-technical Author Summary of your research to make findings accessible to a wide audience that includes both scientists and non-scientists. The Author Summary should immediately follow the Abstract in your revised manuscript. This text is subject to editorial change and should be distinct from the scientific abstract. Please see our author guidelines for more information: <https://eur01.safelinks.protection.outlook.com/?url=https%3A%2F%2Fjournals.plos.org%2Fplosmedicine%2Fs%2Frevising-your-manuscript%23loc-author-summary&data=05%7C01%7Ckejia.hu%40ki.se%7Cfd479f3e66c048e7fa8208dbcbcfa22d%7Cbff7eef1cf4b4f32be3da1dda043c05d%7C0%7C0%7C638327864865895018%7CUnknown%7CTWFpbGZsb3d8eyJWIjoiMC4wLjAwMDAiLCJQIjoiV2luMzIiLCJBTiI6Ik1haWwiLCJXVCI6Mn0%3D%7C3000%7C%7C%7C&sdata=jOEwHNl41ucQPjLbnNBTuJuJLu7ktlacjus6AbrfnLM%3D&reserved=0>

Ideally each sub-heading should contain 2-3 single sentence, concise bullet points containing the most salient points from your study.

In the final bullet point of ‘What Do These Findings Mean?’ Please include the main limitations of the study in non-technical language.

Response: We have anow included an Author Summary in the submission form.

*“Why Was This Study Done?*

- *Mental illness has been associated with a higher risk of cervical cancer and precancerous lesions as well as a lower degree of participation in cervical screening.*
- *Little is known, however, regarding disparities in HPV infection between women with and without mental illness.*

*What Did the Researchers Do and Find?*

- *In a cohort study, we followed all 337,116 women who were at age 30-64, living in Stockholm, and had a negative test result of high-risk HPV during August 2014 to December 2019, to assess the link between mental illness and risk of infection with high-risk HPV.*
- *The absolute infection rate of HPV was 35% higher among women with a specialist diagnosis of mental disorder and 44% higher among women with a filled prescription of psychotropic medications, compared to women without such.*

*What Do These Findings Mean?*

- *Mental illness is associated with an increased infection rate of oncogenic HPV in women.*
- *Refined approaches are needed to facilitate the elimination agenda of cervical cancer among women with mental illness.” (Authors summary, page3 3-4, lines 35-53)*

METHODS:

Please add the statistical method used to your method section.

Response: We have further clarified the statistical method in the Statistical Analysis section.

*“To ensure generalizability of our findings, we first compared the prevalence of mental illness in the study cohort (i.e., women at screening age who were tested negative for high-risk HPV at cohort entry) to the prevalence in the alternative female population at screening age in Stockholm (i.e., entire Stockholm female population after excluding our study cohort). We calculated prevalence through dividing exposed proportion of follow-up time by the total follow-time during the study period of both the study cohort and the alternative female population in Stockholm.*

*Within the study cohort, participant characteristics were summarized by the distribution of person-years among women with or without mental illness, using time-varying exposure and other time-varying covariables. We fitted multivariable Cox proportional hazards model to estimate the relative infection rate of high-risk HPV in relation to diagnosis of mental disorder or use of psychotropic medications, as hazard ratios (HR) and 95% confidence intervals (CI). We reported p values of the coefficient using likelihood ratio test. We also fitted separate Cox models for HPV-16/18 and other high-risk HPV infection, as positivity of different types of HPV might be reported in one HPV test. We employed attained age as the underlying time scale for our analysis. No major violation of the proportional hazard assumption was found after plotting the Schoenfeld residuals over time. Models were implicitly adjusted for age (as age was used as the underlying time scale), and further adjusted for country of birth, educational level, HPV vaccination status, and maternal history of CIN3+. Finally, to examine potentially differential attendance in the subsequent screening between women with or without mental illness, we defined women who ended the follow-up at one screening interval plus six months as loss to follow-up (i.e., missing a subsequent screening) and estimated HR of loss to follow-up in relation to mental illness, using Cox models with the same adjustment.*

*To examine potential effect modification by age, we fitted interaction models by adding an interaction term between age (30-39, 40-49, and 50-64 years) and mental illness. We tested for the statistical significance of the interaction by log-likelihood ratio test. To assess the role of severity of mental illness on HPV infection rate, we performed a sensitivity analysis by analyzing separately women with a specialist diagnosis of mental disorder (regardless of medication use) and women with only filled prescription of psychotropic medications (i.e., without a specialist diagnosis). We further performed a sensitivity analysis by censoring at one screening interval plus 12 instead of six, months since the last negative test for high-risk HPV, to allow more time for delay in attending the next screening. We performed an additional sensitivity analysis by treating data as interval censored, i.e., the event could happen any time between the last negative test and the first positive test, or any time after the last negative test. We fitted Cox models to estimate the HRs and estimated the standard error using bootstrapping.”* (Methods section, pages 8-10, lines 160-194)

Please add the following statement, or similar, to the Methods: ""This study is reported as per the Strengthening the Reporting of Observational Studies in Epidemiology (STROBE) guideline (S1 Checklist).

Response: We have added the statement.

*“This study is reported as per the Strengthening the Reporting of Observational Studies in Epidemiology (STROBE) guideline (S2 Checklist).”* (Methods section, page 10, lines 201-202)

RESULTS:

As above, please provide 95% CIs and p values for all results were appropriate

Response: We have provided 95% CIs and p values throughout the manuscript where appropriate.

ACKNOWLEDGMENTS/ DECLARATIONS

Please remove all statements apart from acknowledgements, author contributions and abbreviations from the end of the main manuscript and include these only in the relevant parts of the manuscript submission form. Funding, competing interest, and data availability will be compiled as metadata.

Response: We have removed these statements from the main manuscript and included them as metadata in the submission form.

REFERENCES:

Please use the "Vancouver" style for reference formatting, and see our website for other reference guidelines <https://eur01.safelinks.protection.outlook.com/?url=https%3A%2F%2Fjournals.plos.org%2Fplosmedicine%2Fs%2Fsubmission-guidelines%23loc-references&data=05%7C01%7Ckejia.hu%40ki.se%7Cfd479f3e66c048e7fa8208dbcbcfa22d%7Cbff7eef1cf4b4f32be3da1dda043c05d%7C0%7C0%7C638327864865895018%7CUnknown%7CTWFpbGZsb3d8eyJWIjoiMC4wLjAwMDAiLCJQIjoiV2luMzIiLCJBTiI6Ik1haWwiLCJXVCI6Mn0%3D%7C3000%7C%7C%7C&sdata=90b60MtYjfepAppiaL8Lw1Ralxs0003ZT0OV2MnJwSk%3D&reserved=0>

Please ensure that in the bibliography up to but no more than 6 author names are listed, followed by et al., in the event that more than 6 authors contribute to an individual study. Journal name abbreviations should be those found in the National Center for Biotechnology Information (NCBI) databases.

Please also ensure that any references to online-only sources include a date of accession, and in the reference list, please convert all italics to plain text.

Response: We have now amended the reference style as requested.

FIGURES:

Figure 2: Please consider avoiding the use of red and green in order to make your figure more accessible to those with colour blindness. Likewise for figure S1.

Response: Thanks for the suggestion. We have now modified Figure 2 and Figure S1 as suggested.

-----------------------------------------------------------

Comments from the reviewers:

Reviewer #1: Thank you for the opportunity to review this timely and important paper on an under-researched area examining the relationship between HPV infection acquisition by women with and without mental illness within the context of an HPV based screening program. This question is pertinent in the context of the global quest for the elimination of cervical cancer.

The paper is well written and concise and fills important gaps in the literature. The figures and tables are well portrayed.

The paper's strengths lie in its robust approach through analysis of participants in randomised implementation trials and Sweden's data linkage capability to link HPV screening results to mental illness diagnoses/use of psychotropic medications, and to HPV vaccination and maternal history of cervical disease.

However, the limitations require further consideration, in particular the choice of a relatively short 6-month period of follow up which inevitably means that a sizeable group of participants with mental illness are deemed 'lost to follow up' which will likely lead to potential underestimation of HPV infection (ie the primary outcome). The relatively sample size relative to the Stockholm population is well made due to the requirement for a negative HPV results to enter the trial and the relatively short follow up period due to the relative recency of the introduction of HPV screening in Sweden.

The approach to defining who has and hasn't got mental illness has been well described in a previous paper.

The approach to inclusion is well described and the end points of participation are clearly defined.

I have some comments and some suggestions for improvement - I also note that I am not an expert statistician/epidemiologist so strongly recommend expert review in these areas given the relative complexity of their methodological approaches.

Response: Thank you for the very positive yet constructive comments. Please find below our point-to-point responses to your specific comments, including the comment related to the relatively short follow-up, where we performed additional analysis using 12-month grace period which showed similar results. The page and line numbers refer to the clean version of the manuscript (.docx).

A couple of general comments:

1.	While not a criticism of the paper the rate of new infections (incidence rate) of 1% seems relatively low?

Response: Thank you for the good comment. In our study, 3,263 of the 337,116 study participants were tested positive for high-risk HPV during the study. The incidence rate is indeed lower than the ones reported in previous studies (i.e., 6-32% during a 12-month follow-up period) (refs 31 and 32 in the manuscript). There are several potential explanations for such a discrepancy.

First, the participants of the present study were required to have a negative test for high-risk HPV according to an HPV screening during 08/2014-12/2019, indicating that this is a relatively low risk group in terms of HPV infection.

Second, previous studies were based on smaller cohorts with closer monitoring of HPV infection (i.e., women were tested every 4 to 6 months). In our study, however, we detected HPV infection primarily through an HPV screening (every 3 years at age 30-49 and every 5 years at age 50-64). As a result, we might have missed HPV infections acquired and cleared between two screening rounds.

Third, as we have a relatively short follow-up (median: 2.21 years; range: 0-5.42 years), many women had not yet had a next screening round.

However, we could not find any other study with a similar setting as ours for comparison, as Stockholm is one of the earliest to implement such screening program.

Regardless, we have now added these as limitations in the Discussion section.

*“First, we could only detect HPV infection through screening and therefore might* *have missed infections acquired and cleared between two screening rounds. Similarly, as the eligible participants of the study had to have participated in HPV screening and have had a negative test result for HPV infection, the study participants were likely at a lower risk of HPV infection compared to other women. As a result of both, the infection rate* *of high-risk HPV infection appeared lower in the present study,* *compared to previous studies* *[31,32]. As these conditions are unlikely differential in relation to the exposure (i.e., mental illness), the results of the present study might most likely be an underestimate of the real association between mental illness and HPV infection.**”* (Discussion section, page 14, lines 317-324)

*“**Third, we had a relatively short follow-up as HPV-based screening was only introduced in 2014 in Stockholm. As a result, the majority (82%) of women in our study did not have had the time to receive another screening. However, Stockholm was among the earliest regions to initiate HPV-based screening in Europe [33], and our study represents the largest study to date with the longest follow-up concerning women regularly screened for HPV infection. Regardless, follow-up studies with longer follow-up or larger sample size (e.g., national data) are needed to validate these findings with better statistical power, especially for subgroup analysis.”* (Discussion section, pages 14-15, lines 328-335)

References

31.	Paul P, Hammer A, Rositch AF, Burke AE, Viscidi RP, Silver MI, et al. Rates of New Human Papillomavirus Detection and Loss of Detection in Middle-aged Women by Recent and Past Sexual Behavior. J Infect Dis. 2021;223(8):1423–32.

32.	Giuliano AR, Harris R, Sedjo RL, Baldwin S, Roe D, Papenfuss MR, et al. Incidence, prevalence, and clearance of type-specific human papillomavirus infections: The Young Women’s Health Study. J Infect Dis. 2002;186(4):462–9.

33.	Maver PJ, Poljak M. Primary HPV-based cervical cancer screening in Europe: implementation status, challenges, and future plans. Clin Microbiol Infect. 2020 May 1;26(5):579–83.

2.	The data outcomes are generally robust and the sample from the randomised trial are generally representative of the national population, however, it could be argued that it could have been preferable to wait until more national HPV data becomes available for analysis to increase the size of some of the groups?

Response: Thanks for the good point. We agree and have now addressed this in the Discussion section.

*“Third, we had a relatively short follow-up as HPV-based screening was only introduced in 2014 in Stockholm…. However, Stockholm was among the earliest regions to initiate HPV-based screening in Europe [33], and our study represents the largest study to date with the longest follow-up concerning women regularly screened for HPV infection.* *Regardless, future studies with longer follow-up or larger sample size (e.g., national data) are needed to validate these findings with better statistical power, especially for subgroup analysis.”* (Discussion section, pages 14-15, lines 328-335)

Reference

33.	Maver PJ, Poljak M. Primary HPV-based cervical cancer screening in Europe: implementation status, challenges, and future plans. Clin Microbiol Infect. 2020 May 1;26(5):579–83.

Specific comments:

3.	The information on page 4/ 5 regarding the randomized implementation trials would benefit from a little more context - e.g. were these whole of Stockholm population trials?

Response: Yes, the trial covered the entire Stockholm population. We have now added more information on page 5.

*“A randomized implementation of HPV-based screening was initiated in the greater Stockholm area in 2012 [14,15], where all women living in the area who were at screening ages were randomly assigned to the invitation of either primary cytology-based screening with HPV test as triage for women with low-grade cytology, or primary HPV-based screening with cytology as triage for women with positive HPV test. The greater Stockholm area has around one million women, representing 20% of the entire female population in Sweden.”* (Methods section, page 6, lines 85-90)

References

14.	Elfström KM, Eklund C, Lamin H, Öhman D, Hortlund M, Elfgren K, et al. Organized primary human papillomavirus–based cervical screening: A randomized healthcare policy trial. PLOS Med. 2021 Aug 1;18(8):e1003748.

15.	Lamin H, Eklund C, Elfström KM, Carlsten-Thor A, Hortlund M, Elfgren K, et al. Randomised healthcare policy evaluation of organised primary human papillomavirus screening of women aged 56–60. BMJ Open. 2017 May 1;7(5):e014788.

4.	A brief explanation about how the screening program is organised and in particular how/when reminders/recalls are sent would be useful (especially considering the point below re the challenges of the very short 6 month time frame for re-screening after which the person is deemed lost to follow up and excluded from the study)?

Response: We have now further clarified the organization of the program.

*“Women are invited by letter to their randomized screening modality when three years (for women at age 30-49) or five years (for women at age 50-64) have passed since their last negative test; reminder invitations are sent every year to women who did not attend after being invited during the last year [14]. ”* (Methods section, page 6, lines 92-95)

Reference

14.	Elfström KM, Eklund C, Lamin H, Öhman D, Hortlund M, Elfgren K, et al. Organized primary human papillomavirus–based cervical screening: A randomized healthcare policy trial. PLOS Med. 2021 Aug 1;18(8):e1003748.

5.	The chosen period of 6 months grace for women to return for their follow up 3 or 5-yearly HPV screening test before being deemed lost to follow up is very short (as noted by the authors) and needs explanation as to why it was chosen rather than, say, 12 months?

This is highly relevant (also noted by the authors) for those groups with certain mental illness diagnoses (particularly those with neurodevelopmental disorders including autism) where multiple barriers may preclude such timely access. While the authors have noted this may lead to an underestimation of HPV incident infection in these groups, this seems to be a major challenge for the central premise of the paper given that these groups may also be more likely to have experienced sexual abuse than the general population which may go un-noted without sufficient time to present for follow up screening since their previous negative test? As also noted this may lead to the lack of statistical power and statistically significant findings for several mental illnesses.

In short, the implications for people with a mental illness diagnosis being less likely to be screened but more likely to acquire HPV would benefit from greater scrutiny in the paper (noting that a focus on timely screening may be most relevant from a policy perspective?)

Response: Thanks for the great comment and good suggestion. We have now added a sensitivity analysis using 12-month grace period to define loss to follow-up and shown the results in Supplemental Table 6. As the reviewer correctly suspected, this added more cases of high-risk HPV infection and improved statistical power, however, the overall associations did not differ greatly from that of the main analysis. We have now described the new analysis in the Methods and Results sections. Please see our response below regarding sexual abuse.

*“We further performed a sensitivity analysis by censoring at one screening interval plus 12, instead of six, months since the last negative test for high-risk HPV, to allow more time for delay in attending the next screening.”* (Methods section, page 9, lines 188-190)

*“Sensitivity analysis using 12 months as the grace period showed similar results as the main analysis (Supplemental Table 6).”* (Results section, page 12, lines 252-253)

**Supplemental Table 6. Adjusted hazard ratios (HRs) with 95% confidence intervals (CIs) of high-risk HPV infection in relation to diagnosis of mental disorder or use of psychotropic medication, using a 12-month grace period to define loss to follow-up**

|  |  | **Any high risk-HPV**^1^ | | | **HPV16/18** | | | **Other high risk-HPV** | | |
| --- | --- | --- | --- | --- | --- | --- | --- | --- | --- | --- |
| Groups | Person-years | No. of infection | Adjusted HR (95%CI)^2^ | P-value | No. of infection | Adjusted HR (95%CI)^2^ | P-value | No. of infection | Adjusted HR (95%CI)^2^ | P-value |
| **Any diagnosis of mental disorder** | | | | | | | | | | |
| No | 682,113 | 2868 | Reference |  | 529 | Reference |  | 2,281 | Reference |  |
| Yes | 113,498 | 673 | 1.34 (1.23-1.46) | p<0.001 | 116 | 1.25 (1.02-1.53) | 0.025 | 545 | 1.37 (1.26-1.50) | p<0.001 |
| **Any diagnosis of psychiatric disorder** | | | | | | | | | | |
| No | 684424 | 2883 | Reference |  | 529 | Reference |  | 2488 | Reference |  |
| Yes | 111188 | 658 | 1.34 (1.23-1.46) | p<0.001 | 116 | 1.29 (1.05-1.57) | 0.013 | 580 | 1.37 (1.25-1.50) | p<0.001 |
| **Any diagnosis of neurodevelopmental disorder** | | | | | | | | | | |
| No | 783873 | 3467 | Reference |  | 639 | Reference |  | 2998 | Reference |  |
| Yes | 11738 | 74 | 1.20 (0.95-1.51) | 0.123 | 6 | 0.53 (0.23-1.18) | 0.118 | 70 | 1.31 (1.03-1.67) | 0.026 |
| **Any use of psychotropic medication** | | | | | | | | | | |
| No | 458163 | 1783 | Reference |  | 337 | Reference |  | 1528 | Reference |  |
| Yes | 337449 | 1758 | 1.43 (1.34-1.53) | p<0.001 | 308 | 1.32 (1.13-1.54) | p<0.001 | 1540 | 1.47 (1.37-1.58) | p<0.001 |

^1^ High-risk HPV includes 14 types: 16, 18, 31, 33, 35, 39, 45, 51, 52, 56, 58, 59, 66, and 68

^2^ Adjusted for age, country of birth, educational level, HPV vaccination status, and maternal history of CIN3+

6.	Another argument that could be considered relates to latency of HPV acquired at an earlier age (eg due to child sexual abuse), whereby the HPV is undetectable at the time of screening (and in this case at the time of the prior screen) and then reactivated later with a decline in immune function and goes on to cause disease - this may be pertinent given the relatively high rates of sexual abuse for some groups under the mental illness umbrella?

Response: Thank you for raising an interesting point. We have now added discussion about child sexual abuse in the Introduction and Discussion sections.

*“Several factors may contribute to the elevated risk of HPV infection among women with mental illness, …, and a higher chance of having experienced sexual abuse [11], compared to their counterparts.”* (Introduction section, page 5, lines 70-73)

*“**In addition, women with mental illnesses are more likely to have experienced adverse life events such as childhood sexual abuse [26],* *a traumatic life event previously shown to be associated with both the development of mental ill health and an elevated risk of HPV infectio**n [11,27]. Although the precise mechanisms remain unknown, immune alterations might have contributed, in acquiring, clearing, and reactivating of HPV infection [26,27].”* (Discussion section, page 13, lines 280-282)

References:

11.	Bulik CM, Prescott CA, Kendler KS. Features of childhood sexual abuse and the development of psychiatric and substance use disorders. Br J Psychiatry. 2001;179(5):444–9.

26.	Nelson CA, Scott RD, Bhutta ZA, Harris NB, Danese A, Samara M. Adversity in childhood is linked to mental and physical health throughout life. BMJ. 2020;371:1–9.

27.	Cao CD, Merjanian L, Pierre J, Balica A. A Discussion of High-Risk HPV in a 6-Year-Old Female Survivor of Child Sexual Abuse. Case Rep Obstet Gynecol. 2017;2017:1–4.

7.	The argument around biological plausibility for increased HPV acquisition due to immune suppression and other mechanisms is well made - ie it is not all behavioural/modifiable?

Response: Thanks for the comment. We have further discussed the possible mechanisms.

*“**For example, it has been suggested that women with mental illness are more likely to contract sexually transmitted diseases, have sexual debut at an early age [8], and smoke [9]. Several illegal drugs, including cannabis, may exhibit immunosuppressive effects, potentially increasing the susceptibility to viral infections [24]. Notably, a higher proportion of individuals with mental illness use these substances compared to the general population [25]. In addition, women with mental illnesses are more likely to have experienced adverse life events such as childhood sexual abuse [26],* *a traumatic life event previously shown to be associated with both the development of mental ill health and an elevated risk of HPV infection [11,27]. Although the precise mechanisms remain unknown, immune alterations might have contributed, in acquiring, clearing, and reactivating of HPV infection [26,27]. Although not all factors are modifiable, targeted interventions are possible and feasible in order to minimize such disparities.”* (Discussion section, pages 12-13, lines 275-285)

Minor points

8.	Page 4 line 38 - the term vulnerable is generally not preferred as it implies weaknesses on the part of the individual rather than society - it could be replaced by a term such as marginalised/under-served?

Response: Thanks for the suggestion. We have changed the term to “marginalized” throughout the manuscript.

9. There is a minor typo dirode

Response: We have corrected this typo – many thanks.

Reviewer #2: Herweijer et al. have conducted a population-based cohort study in Sweden to investigate the incidence of oncogenic HPV infection in women with and without mental illness. The study aimed to determine whether women with mental illness have a higher risk of HPV infection compared to those without mental illness.

Using a cohort of 337,116 women aged 30-64 living in Stockholm, who had previously tested negative for 14 high-risk HPV subtypes in HPV-based screening from August 2014 to December 2019, the researchers identified women with mental illness based on specialist diagnoses of mental disorders or filled prescriptions for psychotropic medication.

The results revealed that women with mental illness had an increased risk of HPV infection compared to women without mental illness. Specifically, those with a specialist diagnosis of mental disorder had a hazard ratio (HR) of 1.35 (95% confidence interval [CI]: 1.23-1.47), while those with filled prescriptions for psychotropic medication had an HR of 1.44 (95% CI: 1.34-1.54). This elevated risk was observed across various mental health conditions, including depression, anxiety, stress-related disorders, and substance-related disorders, as well as among users of antidepressants, anxiolytics, sedatives, and hypnotics. Importantly, these findings held true across different age groups.

However, the study had certain limitations, such as the selection of the female population in Stockholm and the relatively short follow-up period due to the introduction of HPV-based screening in 2014.

In conclusion, this study suggests that women with mental illness are at a higher risk of HPV infection. These findings underscore the need for targeted strategies to address cervical cancer risk in this vulnerable population, aligning with the WHO's agenda for the global elimination of cervical cancer.

The claims are properly placed in the context of the previous literature. The experimental data support the claims. The manuscript is written clearly enough that most of it is understandable to non-specialists. The authors have provided adequate proof for their claims, without overselling them. The authors have treated the previous literature fairly. The paper offers enough details of methodology so that the experiments could be reproduced.

Response: Many thanks for the encouraging comments to our manuscript. Please find below our point-to-point responses to your specific comments. The page and line numbers refer to the clean version of the manuscript (.docx).

Minor revisions

1. Page 4, line 43-44, "It is therefore likely that mental illness influences the risk of cervical cancer development also through other mechanisms like HPV infection."

Unclear. Please reformulate. It is important to note that HPV is the primary cause of cervical cancer in both women with mental illnesses and all other women.

Response: Thank you for the comment and we agree. We have now clarified the sentence as follows:

“This suggests that mental illness likely influences the risk of cervical cancer also through other mechanisms apart from differential screening participation, including modulating the risk of infection with HPV, the primary cause of cervical cancer.” (Introduction section, page 5, lines 65-68)

2. Page 4, line 46-69, add "sexual abuse"

"An elevated risk of acquiring HPV infection is indeed plausible among women with mental illness, due to biological and behavioral factors. For instance, women with mental illness have been suggested to demonstrate abnormal levels of immune biomarkers, engage in riskier sexual behaviors, be more likely to smoke, have limited knowledge of HPV infection, and may also have a higher prevalence of sexual abuse compared to other women."

Response: Thanks for the good point. We have added sexual abuse in this paragraph and added the references you listed.

*“Several factors may contribute to the elevated risk of HPV infection among women with mental illness, …, and a higher chance of having experienced sexual abuse [11], compared to their counterparts.”* (Introduction section, page 5, lines 70-73)

Reference:

11.	Bulik CM, Prescott CA, Kendler KS. Features of childhood sexual abuse and the development of psychiatric and substance use disorders. Br J Psychiatry. 2001;179(5):444–9.

3. Alternative introduction:

"Cervical cancer, primarily caused by oncogenic human papillomavirus (HPV) infections on the cervix, is preventable through HPV vaccination and cervical screening, facilitating early detection and treatment of precancerous lesions. In 2020, the World Health Organization (WHO) launched efforts to eliminate cervical cancer as a public health issue. However, women with mental illness present a unique challenge to this agenda, as they not only face an increased risk of cervical cancer but also tend to have reduced participation in cervical screening.

This heightened risk among women with mental illness cannot be solely attributed to lower screening rates. Even among those who participate in screening, women with specialist diagnoses of mental disorders still carry a twofold risk of developing cervical precancerous lesions. This suggests that mental illness likely influences cervical cancer risk through other mechanisms, including the potential impact of HPV infection.

Several factors contribute to the plausible elevated risk of HPV infection among women with mental illness, encompassing abnormal levels of immune biomarkers, engagement in riskier sexual behaviors, higher smoking rates, limited knowledge about HPV infection, and a potentially higher incidence of sexual abuse compared to their counterparts. Despite these concerns, population-based studies examining HPV infection disparities between women with and without mental illness remain limited.

To address this gap, we conducted a study using data from a substantial prospective cohort of women participating in HPV-based cervical screening in Stockholm, Sweden. Our hypothesis was that women with mental illness would exhibit an increased risk of HPV infection."

Response: We have modified the introduction according to your edits.

*“Cervical cancer, primarily caused by infection with oncogenic human papillomavirus (HPV) on the cervix [1], is preventable through HPV vaccination, which prevents HPV infection, and cervical screening, which enables early detection and treatment of cervical precancerous lesions. In 2020, the World Health Organization (WHO) launched efforts to accelerate the elimination of cervical cancer as a public health issue [2]. However, women with mental illness present a unique challenge to this agenda, as they not only face an increased risk of cervical cancer but also tend to have reduced participation in cervical screening [3–6].*

*This heightened risk among women with mental illness cannot be solely attributed to lower screening rates. Even among those who participate in screening, women with specialist diagnoses of mental disorders still carry a twofold risk of developing cervical precancerous lesions [3].* *This suggests that mental illness likely influences the risk of cervical cancer also through other mechanisms apart from differential screening participation, including modulating the risk of infection with HPV, the primary cause of cervical cancer.*

*Several factors may contribute to the elevated risk of HPV infection among women with mental illness, encompassing abnormal levels of immune biomarkers [7], engagement in more risky sexual behaviors [8], higher smoking rate [9], limited knowledge about HPV infection [10], and a higher chance of having experienced sexual abuse* *[11], compared to their counterparts. Despite these concerns, population-based studies examining HPV infection disparities between women with and without mental illness remain limited.*

*To address this gap, we conducted a study using data from a substantial prospective cohort of women participating in HPV-based cervical screening in Stockholm, Sweden. Our hypothesis was that women with mental illness would exhibit an increased risk of HPV infection.”* (Introduction section, page 5, lines 55-79)

4. Page 5, line 64-65, "The trial initially focused on women aged 56-60 but later expanded into a comprehensive randomized implementation that encompassed women aged 30-64 starting from August 2014."

Response: We have made the change as you suggested in the Methods section, page 6, lines 90-92.

5. Page 5, line 67-68, "As a result, beginning in 2017, all women between the ages of 30 and 64 in the greater Stockholm area underwent primary screening using HPV-based testing."

Response: We have made the change as you suggested in the Methods section, page 6, lines 98-100.

6. Page 5, line 68-69, "The screening test covers 14 high-risk HPV types, including HPV-16, 18, 31, 33, 35, 39, 45, 51, 52, 56, 58, 59, 66, and 68."

Response: We have made the change as you suggested in the Methods section, page 6, lines 100-101.

7. Page 7, line 140, "We employed attained age as the fundamental time scale for our analysis."

Response: We have made the change as you suggested in the Methods section, page 8, lines 173-174.

8. Page 10, line 227, add "A significant number of women with mental illnesses have experienced childhood sexual abuse. This traumatic experience can lead to the development of mental health disorders and is also associated with an elevated risk of both HPV infection and cervical cancer."

Bulik, C., Prescott, C., & Kendler, K. (2001). Features of childhood sexual abuse and the development of psychiatric and substance use disorders. The British Journal of Psychiatry, 179(5), 444-449. doi:10.1192/bjp.179.5.444

Cao CD, Merjanian L, Pierre J, Balica A. A Discussion of High-Risk HPV in a 6-Year-Old Female Survivor of Child Sexual Abuse. Case Rep Obstet Gynecol. 2017;2017:6014026. doi: 10.1155/2017/6014026. Epub 2017 May 23. PMID: 28620555; PMCID: PMC5460386.

Response: Thanks. We have added the sentence and the references you listed.

*“In addition, women with mental illnesses are more likely to have experienced adverse life events such as childhood sexual abuse [26], a traumatic life event previously shown to be associated with both the development of mental ill health and an elevated risk of HPV infection [11,27]. Although the precise mechanisms remain unknown, immune alterations might have contributed, in acquiring, clearing, and reactivating of HPV infection [26,27].”* (Discussion section, page 13, lines 280-282)

References:

11.	Bulik CM, Prescott CA, Kendler KS. Features of childhood sexual abuse and the development of psychiatric and substance use disorders. Br J Psychiatry. 2001;179(5):444–9.

26.	Nelson CA, Scott RD, Bhutta ZA, Harris NB, Danese A, Samara M. Adversity in childhood is linked to mental and physical health throughout life. BMJ. 2020;371:1–9.

27.	Cao CD, Merjanian L, Pierre J, Balica A. A Discussion of High-Risk HPV in a 6-Year-Old Female Survivor of Child Sexual Abuse. Case Rep Obstet Gynecol. 2017;2017:1–4.

9. Page 10, line 227, "Several illegal drugs, including cannabis, may exhibit immunosuppressive effects, potentially increasing the susceptibility to viral infections. Notably, a higher proportion of individuals with mental illness use these substances compared to the general population."

Response: We have made the change as you suggested in the Discussion section, page 13, lines 277-280.

10. Page 12, line 276, "Fortunately, self-collected HPV tests have been introduced to enhance cervical screening since July 2020 in Sweden."

Response: We have made the change as you suggested in the Discussion section, page 15, lines 347-348.

11. Page 20-22, In Table 2 and Table 3, for enhanced readability, please use thousand separators in large numbers (e.g., 668494 => 668,494).

Response: We have made the change as you suggested in Table 2, Table 3, and all other tables. Thank you for your thorough edits to the manuscript.

Reviewer #3: Alex McConnachie, Statistical Review

This review looks at the statistical elements of the paper by Herweijer et al, which presents data from linked routine datasets to assess the association between mental illness (hospital diagnosis, or determined by prescription history), and high-risk HPV infection, detected through screening in Stockholm, Sweden.

On the face of it, the methods look very good, with Cox models used to assess associations whilst adjusting for potential confounders. The PH assumption is checked; various subgroup and sensitivity analyses are reported, and the results are presented quite clearly, and interpreted appropriately.

However, there are one or two issues with the analysis that I can see.

Response: Thanks for the positive comments on our statistical analysis. Please find below our point-to-point responses to your specific comments. The page and line numbers refer to the clean version of the manuscript (.docx).

1. The study cohort is defined as those women living in the study area between 01/08/2014 and 31/12/2019, aged 30-64, with a negative test for HPV during this period. Date of entry to the cohort is defined as the latest of their first negative test, their 30th birthday, or 01/08/2014. Given the cohort definition, is this essentially the same as saying that women enter the cohort at their first negative test?

Since someone must have a negative test during the time window, their first negative test must come after 01/08/2014, so it seems redundant to include 01/08/2014 in the definition. For anyone who turns 30 during the study period, their HPV status at that point is unknown, regardless of whether they had a previous test result, so it would make more sense to make the start date equal to the first negative test during the study period after their 30th birthday.

Response: Thanks for the opportunity to clarify. The HPV-based screening is offered to women at age 30-64 since August 2014. However, as the trial has started since 2012 already for women at age 56-60 and women could also receive an HPV test outside the program, some women could have had HPV tests before August 2014 or before age 30. By defining the start of follow-up as the latest of their first negative test, their 30th birthday, or 01/08/2014, we ensured to follow women only after the start of full-scale screening program. We have clarified this in the manuscript.

*“To ensure that women were followed after the start of the full-scale screening program, women entered the cohort on the date of their first negative HPV test, 30th birthday, or August 1st, 2014, whichever occurred last.”* (Methods section, pages 6-7, lines 109-111).

2. There is a similar problem in defining the date of censoring. This is defined as the earliest of their 65th birthday, emigration (from Stockholm, or from Sweden), death, 31/12/2019, or one screening interval plus 6 months after their last HPV test (defined as lost to follow-up). This is a major issue; for all those people who are censored, the Cox model will assume that the event (HPV infection) has not occurred, when in fact, the infection status of people who are censored is unknown. They may or may not have been infected since their last negative test result.

Response: We agree with the reviewer’s concern. As the reviewer suggested, we have now added an analysis by treating the data as interval censored and present the new results in Supplemental Table 7. As correctly guessed by the reviewer, this analysis led to slightly stronger results as those of the main analysis. We have now described the new analysis in the Methods and Results sections.

*“We performed an additional sensitivity analysis by treating data as interval censored, i.e., the infection could happen any time between the last negative test and the first positive test, or any time after the last negative test. We fitted Cox models to estimate the HRs and estimated the standard errors using bootstrapping.”* (Methods section, pages 9-10, lines 190-194)

*“Results from the interval censored data analysis were in general similar to the ones obtained in the main analysis, although the estimated HRs were slightly greater than the ones obtained in the main analysis (Supplemental Table 7).”* (Results section, page 12, lines 253-255)

**Supplemental Table 7. Adjusted hazard ratios (HRs) with 95% confidence intervals (CIs) of high-risk HPV infection in relation to diagnosis of mental disorder or use of psychotropic medication, using interval censored data**

| **Exposure** | **Adjusted hazard ratios (HRs)** ^1^ | **P value** |
| --- | --- | --- |
| Any diagnosis of mental disorder | 1.45 (1.34, 1.57) | <0.001 |
| Any diagnosis of psychiatric disorder | 1.44 (1.32, 1.57) | <0.001 |
| Depression | 1.39 (1.23, 1.59) | <0.001 |
| Anxiety | 1.40 (1.24, 1.58) | <0.001 |
| Stress-related disorder | 1.47 (1.31, 1.66) | <0.001 |
| Alcohol-related disorder | 2.02 (1.63, 2.50) | <0.001 |
| Tobacco-related disorder | 1.43 (0.08, 24.17) | 0.806 |
| Substance abuse ^2^ | 1.92 (1.38, 2.68) | <0.001 |
| Psychotic disorder | 0.97 (0.56, 1.69) | 0.917 |
| Any diagnosis of neurodevelopmental disorder | 1.47 (1.19, 1.83) | <0.001 |
| Attention-deficit hyperactivity disorder (ADHD) | 1.53 (1.20, 1.95) | <0.001 |
| Intellectual disability | 2.03 (1.07, 3.86) | 0.029 |
| Autism | 0.69 (0.33, 1.44) | 0.329 |
| Any use of psychotropic medication | 1.67 (1.55, 1.79) | <0.001 |
| Sedatives/hypnotics | 1.59 (1.48, 1.72) | <0.001 |
| Anxiolytics | 1.57 (1.45, 1.69) | <0.001 |
| Antidepressants | 1.45 (1.35, 1.55) | <0.001 |
| Antipsychotics | 1.28 (1.09, 1.49) | 0.002 |

HR-HPV: high-risk HPV, including 14 types: 16, 18, 31, 33, 35, 39, 45, 51, 52, 56, 58, 59, 66, and 68.

^1^ Adjusted for age, country of birth, educational level, HPV vaccination status, and maternal history of CIN3+

^2^: Tobacco and alcohol-related disorders are excluded.

We have also added a sensitivity analysis using 12-month grace period to define loss to follow-up and shown the results in Supplemental Table 6. Again, as the reviewer correctly suspected, this added more cases of high-risk HPV infection and improved statistical power. However, the overall associations did not differ greatly from that of the main analysis. We have now described the new analysis in the Methods and Results sections.

*“We further performed a sensitivity analysis by censoring at one screening interval plus 12, instead of six, months since the last negative test for high-risk HPV, to allow more time for delay in attending the next screening.”* (Methods section, page 9, lines 188-190)

*“Sensitivity analysis using 12 months as the grace period showed similar results as the main analysis (Supplemental Table 6).”* (Results section, page 12, lines 252-253)

**Supplemental Table 6. Adjusted hazard ratios (HRs) with 95% confidence intervals (CIs) of high-risk HPV infection in relation to diagnosis of mental disorder or use of psychotropic medication, using 12-month grace period to define loss to follow-up**

|  |  | **Any high risk-HPV**^1^ | | | **HPV16/18** | | | **Other high risk-HPV** | | |
| --- | --- | --- | --- | --- | --- | --- | --- | --- | --- | --- |
| Groups | Person-years | No. of infection | Adjusted HR (95%CI)^2^ | P-value | No. of infection | Adjusted HR (95%CI)^2^ | P-value | No. of infection | Adjusted HR (95%CI)^2^ | P-value |
| **Any diagnosis of mental disorder** | | | | | | | | | | |
| No | 682,113 | 2868 | Reference |  | 529 | Reference |  | 2,281 | Reference |  |
| Yes | 113,498 | 673 | 1.34 (1.23-1.46) | p<0.001 | 116 | 1.25 (1.02-1.53) | 0.025 | 545 | 1.37 (1.26-1.50) | p<0.001 |
| **Any diagnosis of psychiatric disorder** | | | | | | | | | | |
| No | 684424 | 2883 | Reference |  | 529 | Reference |  | 2488 | Reference |  |
| Yes | 111188 | 658 | 1.34 (1.23-1.46) | p<0.001 | 116 | 1.29 (1.05-1.57) | 0.013 | 580 | 1.37 (1.25-1.50) | p<0.001 |
| **Any diagnosis of neurodevelopmental disorder** | | | | | | | | | | |
| No | 783873 | 3467 | Reference |  | 639 | Reference |  | 2998 | Reference |  |
| Yes | 11738 | 74 | 1.20 (0.95-1.51) | 0.123 | 6 | 0.53 (0.23-1.18) | 0.118 | 70 | 1.31 (1.03-1.67) | 0.026 |
| **Any use of psychotropic medication** | | | | | | | | | | |
| No | 458163 | 1783 | Reference |  | 337 | Reference |  | 1528 | Reference |  |
| Yes | 337449 | 1758 | 1.43 (1.34-1.53) | p<0.001 | 308 | 1.32 (1.13-1.54) | p<0.001 | 1540 | 1.47 (1.37-1.58) | p<0.001 |

^1^ High-risk HPV includes 14 types: 16, 18, 31, 33, 35, 39, 45, 51, 52, 56, 58, 59, 66, and 68

^2^ Adjusted for age, country of birth, educational level, HPV vaccination status, and maternal history of CIN3+

3. The authors look at the probability of loss to follow-up and show that those with prior mental illness are more likely to exit the study in this way, and suggest this may lead to underestimation of the association of interest. This is true, but the same issue will apply to all the other forms of censoring. In the model, anyone who is censored is contributing time at risk, during which it is assumed that there was no infection. In reality, the last date at which it is known that there has been no HPV infection is the date of the last negative test.

Response: We defined loss to follow-up as six months plus the scheduled next screening round in the main analysis. For other forms of censoring (emigration out of Sweden, domestic emigration out of Stockholm, death, or December 31st, 2019), we did not perform such analysis as we have no strong evidence to suspect these forms of censoring are differential in relation to mental illness.

We do however agree with the reviewer that the last date at which it is known that there has been no HPV infection is the date of the last negative test and have now performed an additional analysis using interval censoring data. Please see the responses above and below.

4. Also, the current analysis treats the date of the first positive test as the event date. The analysis is therefore estimating the association between mental illness and the detection of HPV infection, as part of the screening programme. In reality, a positive test indicates that infection occurred at some point since the last negative test. Therefore, the data are interval censored: a negative test indicates that no infection occurred since the previous negative test (though this is probably an assumption, if people can become infected and then clear the infection); a positive test indicates that an infection has occurred since the last negative test.

Therefore, I suggest that the analysis should be redone, defining the date of entry to the cohort as the date of the first negative test, and the date of censoring as the date of the last negative test, and treating the data as interval censored. I believe this will affect the time at risk, but should have no impact on the number of events, so it may have limited impact on the power of the analysis. It may not even affect the overall conclusions very much, but it could.

Response: We agree with the reviewer’s great suggestion and performed analysis on interval censored data as a sensitivity analysis. The estimated hazard ratios are slightly greater than those in the main analysis, except for tobacco-related disorder, psychotic disorder, and autism, which were not statistically significant in the main analysis. We have described this new analysis in the Methods and Results sections and added the results in Supplemental Table 7.

*“We performed an additional sensitivity analysis by treating data as interval censored, i.e., the infection could happen any time between the last negative test and the first positive test, or any time after the last negative test. We fitted Cox models to estimate the HRs and estimated the standard errors using bootstrapping.”* (Methods section, pages 9-10, lines 190-194)

*“Results from interval censored data analysis were similar to the ones obtained in the main analysis, although the estimated HRs were slightly greater than the ones obtained in the main analysis (Supplemental Table 7).”* (Results section, page 12, lines 253-255)

**Supplemental Table 7. Adjusted hazard ratios (HRs) with 95% confidence intervals (CIs) of high-risk HPV infection in relation to diagnosis of mental disorder or use of psychotropic medication, using interval censored data**

| **Exposure** | **Adjusted hazard ratios (HRs)** ^1^ | **P value** |
| --- | --- | --- |
| Any diagnosis of mental disorder | 1.45 (1.34, 1.57) | <0.001 |
| Any diagnosis of psychiatric disorder | 1.44 (1.32, 1.57) | <0.001 |
| Depression | 1.39 (1.23, 1.59) | <0.001 |
| Anxiety | 1.40 (1.24, 1.58) | <0.001 |
| Stress-related disorder | 1.47 (1.31, 1.66) | <0.001 |
| Alcohol-related disorder | 2.02 (1.63, 2.50) | <0.001 |
| Tobacco-related disorder | 1.43 (0.08, 24.17) | 0.806 |
| Substance abuse ^2^ | 1.92 (1.38, 2.68) | <0.001 |
| Psychotic disorder | 0.97 (0.56, 1.69) | 0.917 |
| Any diagnosis of neurodevelopmental disorder | 1.47 (1.19, 1.83) | <0.001 |
| Attention-deficit hyperactivity disorder (ADHD) | 1.53 (1.20, 1.95) | <0.001 |
| Intellectual disability | 2.03 (1.07, 3.86) | 0.029 |
| Autism | 0.69 (0.33, 1.44) | 0.329 |
| Any use of psychotropic medication | 1.67 (1.55, 1.79) | <0.001 |
| Sedatives/hypnotics | 1.59 (1.48, 1.72) | <0.001 |
| Anxiolytics | 1.57 (1.45, 1.69) | <0.001 |
| Antidepressants | 1.45 (1.35, 1.55) | <0.001 |
| Antipsychotics | 1.28 (1.09, 1.49) | 0.002 |

HR-HPV: high-risk HPV, including 14 types: 16, 18, 31, 33, 35, 39, 45, 51, 52, 56, 58, 59, 66, and 68.

^1^ Adjusted for age, country of birth, educational level, HPV vaccination status, and maternal history of CIN3+

^2^: Tobacco and alcohol-related disorders are excluded.

Given the fact that the analysis using interval censored data needs to use future information (i.e., loss to follow-up) to define end of follow-up (i.e., date of last negative test), we are a little reluctant to use this as our main analysis. We are however happy to reconsider our position if the reviewer would disagree with us. Regardless, we have discussed this issue in the Discussion section.

*“Lastly, we detected HPV infection through a positive HPV test, based on HPV screening. In reality, the infection could have happened any time between the last negative test and the first positive test. To alleviate this concern, we conducted a sensitivity analysis by treating data as interval censored and obtained similar, or slightly stronger, results.”* (Discussion section, page 15, lines 335-338)

Other comments

5. If age is the time scale for analysis, why do the models adjust for age?

Response: The time scale is implicitly adjusted in the model by using it as the underlying time scale and we did not additionally adjust for it. We have now clarified this.

*“Models were implicitly adjusted for age (as age was used as the underlying time scale), and further adjusted for country of birth, educational level, HPV vaccination status, and maternal history of CIN3+.”* (Methods section, page 9, lines 175-177)

6. In Table 1, is the data for the Greater Stockholm region actually the whole population, or the whole population minus the study cohort? Would it better for the two columns to show distinct populations?

Response: We have now presented two distinct cohorts in Table 1 and further described this in the Methods and Results sections.

*“To ensure generalizability of our findings, we first compared the prevalence of mental illness in the study cohort (i.e., women at screening age who tested negative for high-risk HPV at cohort entry) to the prevalence in the alternative female population at screening age in Stockholm (i.e., entire Stockholm female population after excluding our study cohort). We calculated prevalence through dividing exposed proportion of follow-up time by the total follow-time during the study period of both the study cohort and the alternative female population in Stockholm.”* (Methods section, page 8, lines 160-165)

*“The proportion of person-time exposed to a specialist diagnosis of mental disorder was slightly lower in the study cohort compared with the alternative general female population in Stockholm (14.20% and 16.40%). A similar pattern was found for use of psychotropic medications (42.37% and 44.41%) (Table 1).”* (Results section, page 10, lines 206-209)

**Table 1. Prevalence of mental illness in the study cohort and the alternative female population of the greater Stockholm region**

| Group | **Proportion of mental illness (%)** | |
| --- | --- | --- |
|  | **Study cohort** | **Alternative cohort in Greater Stockholm region** |
| **Any diagnosis of mental disorder** | 14.20 | 16.40 |
|  |  |  |
| **Any diagnosis of psychiatric disorder** | 13.91 | 15.96 |
| Depression | 6.78 | 8.02 |
| Anxiety | 6.62 | 7.63 |
| Stress-related disorder | 5.74 | 6.10 |
| Substance abuse disorder^1^ | 2.33 | 3.45 |
| Psychotic disorder | 0.72 | 1.11 |
|  |  |  |
| **Any diagnosis of neurodevelopmental disorder** | 1.46 | 2.07 |
| Attention-deficit hyperactivity disorder (ADHD) | 1.22 | 1.63 |
| Intellectual disability | 0.12 | 0.26 |
| Autism | 0.33 | 0.48 |
|  |  |  |
| **Any use of psychotropic medication** | 42.37 | 44.41 |
| Hypnotics and sedatives | 24.73 | 26.91 |
| Anxiolytics | 25.12 | 27.16 |
| Antidepressants | 26.99 | 29.21 |
| Antipsychotics | 3.50 | 4.81 |

^1^ Substance abuse disorder includes alcohol-related, tobacco-related, and other substance abuse disorders.

7. In Supplemental Table 4, data is reportedly shown by "age at infection". Is this correct, assuming not everyone has an infection?

Response: Thank you very much for picking up on that. Supplemental Table 4 shows the hazard ratios of high-risk HPV infection in relation to diagnosis of mental disorder or use of psychotropic medication, by attained age, and not age at infection. We have now clarified this in the Supplemental Table 4.
