# Supplementary material for: Incidence of oncogenic HPV infection in women with and without mental illness: A population-based cohort study in Sweden
Source: PLoS Med. 2024 Mar 25;21(3):e1004372. doi: 10.1371/journal.pmed.1004372 (PMC11259452; doi:10.1371/journal.pmed.1004372)
Supplement: Attachment — Submitted filename: Response_to_reviewers.docx [file pmed.1004372.s011.docx]

Manuscript reference number: PMEDICINE-D-23-02359R2

Dear Dr. Janin,

Thank you for the opportunity to submit a further revised version of our manuscript "Incidence of oncogenic HPV infection in women with and without mental illness: a population-based cohort study in Sweden" (PMEDICINE-D-23-02359R3) to PLOS Medicine.

We appreciate greatly the constructive comments from the editors and the statistical reviewer and have now addressed all comments point-by-point and accordingly made changes to the manuscript. We hope that you will find this revised version acceptable for publication.

Yours sincerely,

Kejia Hu, MD PhD

Institute of Environmental Medicine

Karolinska Institutet

Nobels väg 13 | 171 77 Stockholm, Sweden
+46 76 081 07 52
[kejia.hu@ki.se](mailto:kejia.hu@ki.se)

-----------------------------------------------------------

Requests from the editors:

The editorial team’s view aligns with those of the statistical reviewer. Please carefully revise your manuscript and ensure you implement the comments by the statistical reviewer in full.

Response: Thank you for evaluating our work. We have now carefully revised the manuscript and fully implemented the comments made by the statistical reviewer.

For your data availability statement, you write: “The data underlying the results presented in the study are available from Statistics Sweden (<https://eur01.safelinks.protection.outlook.com/?url=http%3A%2F%2Fwww.scb.se%2Fen&data=05%7C02%7Ckejia.hu%40ki.se%7Ce8e8054438a6423f0e3208dbfe91d0f8%7Cbff7eef1cf4b4f32be3da1dda043c05d%7C0%7C0%7C638383674465632304%7CUnknown%7CTWFpbGZsb3d8eyJWIjoiMC4wLjAwMDAiLCJQIjoiV2luMzIiLCJBTiI6Ik1haWwiLCJXVCI6Mn0%3D%7C3000%7C%7C%7C&sdata=vaWup5PcsJrGt65JVtphEQTZ5A86wWf3jN42kIUjpL0%3D&reserved=0>) and The National Board of Health and Welfare (<https://eur01.safelinks.protection.outlook.com/?url=http%3A%2F%2Fwww.socialstyrelsen.se%2Fen&data=05%7C02%7Ckejia.hu%40ki.se%7Ce8e8054438a6423f0e3208dbfe91d0f8%7Cbff7eef1cf4b4f32be3da1dda043c05d%7C0%7C0%7C638383674465632304%7CUnknown%7CTWFpbGZsb3d8eyJWIjoiMC4wLjAwMDAiLCJQIjoiV2luMzIiLCJBTiI6Ik1haWwiLCJXVCI6Mn0%3D%7C3000%7C%7C%7C&sdata=LguygQlb4P0zqwr9gXX%2FpVjD4obP5SnGFmahzO%2FJxIs%3D&reserved=0>). Updated contact information to access the data can be found through the website.” - please add the contact details to your statement.

Response: We have now added the contact details from both data sources in the statement as below.

“The data underlying the results presented in the study are available from Statistics Sweden ([www.scb.se/en](http://www.scb.se/en). Contact details: Email [scb@scb.se](mailto:scb@scb.se), Telephone +46 10 479 40 00) and The Swedish National Board of Health and Welfare ([www.socialstyrelsen.se/en](http://www.socialstyrelsen.se/en). Contact details: Email [socialstyrelsen@socialstyrelsen.se](mailto:socialstyrelsen@socialstyrelsen.se), Telephone +46 (0)75 247 30 00). Updated contact information to access the data can be found through the websites.”

You also write: “The raw datasets are however unavailable for sharing because of privacy policies and regulations in Sweden.” We appreciated there may be legal restriction the prevent you from sharing raw identifiable data. Is it possible to share the de-identified data? And if not - please also add why not.

Response: We have now added further information about the reason why the individual-level data used in the present study cannot be shared. The main reason is that our data are pseudo-anonymized, rather than de-identified, as there is a key between study ID and the individual personal identification number. Although the key file is not available to the researchers, it is technically still possible to identify the individuals unless the key file is destroyed. A second reason is that, given the large number of variables we used in this study, it is also theoretically possible to identify some individuals with different combinations of these variables. We have now modified the statement as below.

“The raw datasets are however unavailable for sharing because of privacy policies and regulations in Sweden. Researchers may however contact the above data holders and apply for the use of such data in their research.”

We find the explanation regarding the Lancet paper satisfactory, although we think this needs expanding in the manuscript itself to be completely transparent regarding data overlap

Response: We have now further expanded the description of the data overlap in the manuscript.

*“This is in line with our previous study [3] including a population of 4,112,598 women born during 1940-1995 who ever resided in Sweden during 1968-2018, showing an increased risk of precancerous cervical lesions among both groups of women. The 337,116 women we included in this study were at age 30-64, ever lived in Stockholm, and had a negative HPV test, during 08/2014-12/2019. Therefore, the population in this study is a subset of the previous study.”* (Discussion section, pages 12-13, lines 270-275 in the clean version of the manuscript)

As a last point, to help us extend the reach of your research, please provide any X (formerly known as Twitter) handle(s) that would be appropriate to tag, including your own, your co-authors’, your institution, funder, or lab. Please enter in the submission form any handles you wish to be included when we post about this paper.

Response: We do not wish to include any handles in the submission.

Feel free to contact me directly at [kjanin@plos.org](mailto:kjanin@plos.org) if you have any questions about the above,

-----------------------------------------------------------

Comments from the reviewers:

Reviewer #2: All comments have been addressed. The response to the comments is adequate and improved the manuscript.

Response: Thank you for the encouraging words.

Reviewer #3: Alex McConnachie, Statistical Review

I thank the authors for their responses to my previous comments.

Response: Thank you. Please find below our point-to-point responses to your additional comments. The page and line numbers refer to the clean version of the manuscript (.docx).

Some sensitivity analyses have been added. The interval censored analysis shows stronger associations, but the description in the text was slightly confusing. To say that infection could happen "…any time after the last negative test" is strange, since the time after the last negative test should not be used in an interval censored analysis. In fact, I would argue that this observation period should not be used in any analysis of infections, because the infection status at the end of this period is unknown.

Response: Thanks for pointing this out. We fully agree that the time after the last negative test should not be used in an interval censored analysis. We have now carefully reflected on our analyses and apologize that we did not explain them clearly.

Firstly, in the interval censored analysis, we limited the population to women with at least two tests, with the first being a negative test. We then started the follow-up since the first test (negative) and ended the follow-up with the first positive test or the last test (negative).

If the follow-up ended with a positive test, the person is interval censored, i.e., the infection happened between this positive test (coded as the upper end of the interval) and the preceding negative test (coded as the lower end of the interval).

If the follow-up ended with a negative test, the person is right censored, i.e., the infection could have happened after this negative test, but we were not able to observe it. We coded this negative test as the lower end of the interval, whereas the upper end of the interval was coded as missing value (NA).

We used function ic_sp in R package icenReg to fit Cox models for this analysis.

We have now further clarified this in the manuscript.

*“To ensure that there was no undetected HPV status during the follow-up, we, in a subset cohort, performed a secondary analysis by treating data as interval censored, i.e., the infection could have happened any time before a positive test since the preceding negative test. In this analysis, we included only women with at least two tests, with the first being a negative test. We started the follow-up from the negative test and ended the follow-up with the first positive test, or the last negative test if there was no positive test. We fitted Cox models* *to estimate the HRs and estimated the standard errors using bootstrapping.”* (Methods section, pages 9-10, lines 190-196)

This also highlights the issue of the period before the first negative test. I do not believe that this should be considered as part of the study. I would hope that this period is excluded from the interval censored analysis, at least. Can the authors confirm that the interval censored analysis only uses periods of observation that start and end with an HPV test?

Response: Yes, we confirm that the interval censored analysis was limited only to women with at least two tests and the follow-up started and ended with an HPV test. Please see the above response.

This raises the question of how many tests people had during the study period. I could be wrong, but I do not recall seeing this information presented in the paper. Logically, only those women with at least two tests (the first being negative) should be included in analyses of HPV infection incidence.

Response: Yes, only those with at least two tests were included in the interval censored analysis. Please see the above response.

Another analysis has been added defining loss to follow-up as one screening period plus 12-months, rather than 6 months, but I feel this is missing the point. The issue is that after the last HPV test, there is no information about HPV status. By censoring at some point after the last negative test (wherever that is), it is assumed that the individual remains HPV-negative during that time. As far as I can see, the only sensible censoring point is the date of the last negative test.

Saying that, the period after the last negative test is of interest for the analysis of loss to follow-up.

Response: We thank the reviewer for understanding our reasoning. We performed this analysis as requested by a reviewer. Indeed, this analysis has very different assumption than the interval-censored analysis, namely that we assumed that individuals remained HPV-negative until a positive test or end of follow-up. In the interval-censored analysis we limited the population to women with at least two tests and disregarded the follow-up time after the last negative test.

However, this analysis is still important as 1) it analyzed the entire screening population (rather than only women with at least two tests) and 2) as the reviewer very correctly pointed out, it provided the opportunity to demonstrate the differential adherence to HPV screening between groups. We have now discussed this in the manuscript.

*“First, we could only detect HPV infection through screening and therefore might have missed infections acquired and cleared between two screening rounds. Similarly, as the eligible participants of the study had to have participated in HPV screening and have had a negative test result for HPV infection, the study participants were likely at a lower risk of HPV infection compared to other women. As a result of both, the infection rate of high-risk HPV infection appeared lower in the present study, compared to previous studies* *[31,32]. As these conditions are unlikely differential in relation to the exposure (i.e., mental illness), the results of the present study might most likely be an underestimate of the real association between mental illness and HPV infection**.”* (Discussion section, page 14, lines 322-329)

*“Lastly, we detected HPV infection through a positive HPV test. Although we, in the main analyses, assumed that individuals remained HPV-negative until a positive test or end of follow-up (in case of no positive test), in reality, the infection could have happened any time before a positive test (since the preceding negative test) or before the end of follow-up (since the last negative test). To address this, we conducted a secondary analysis on women with at least two tests by treating data as interval censored, which showed similar or slightly stronger results. However, the main analysis was important as it provided the opportunity to study the entire sample of the present study, instead of only women with at least two HPV tests during the study, and the differential adherence to HPV screening in relation to mental illness (i.e., showing that women with mental illness were less adherent to HPV screening than other women).”* (Discussion section, page 15, lines 340-349)
