# Supplementary material for: Incidence of oncogenic HPV infection in women with and without mental illness: A population-based cohort study in Sweden
Source: PLoS Med. 2024 Mar 25;21(3):e1004372. doi: 10.1371/journal.pmed.1004372 (PMC11259452; doi:10.1371/journal.pmed.1004372)
Supplement: Attachment — Submitted filename: Response_to_reviewer_editor.docx [file pmed.1004372.s012.docx]

Manuscript reference number: PMEDICINE-D-23-02359R3

Dear Dr. Janin,

Thank you for the opportunity to submit a further revised version of our manuscript "Incidence of oncogenic HPV infection in women with and without mental illness: a population-based cohort study in Sweden" (PMEDICINE-D-23-02359R4) to PLOS Medicine.

We appreciate greatly the constructive comments from the editors and the statistical reviewer and have now addressed the remaining comments point-by-point and accordingly made changes to the manuscript. We hope that you will find this revised version acceptable for publication.

Yours sincerely,

Kejia Hu, MD PhD

Institute of Environmental Medicine

Karolinska Institutet

Nobels väg 13 | 171 77 Stockholm, Sweden
+46 76 081 07 52
[kejia.hu@ki.se](mailto:kejia.hu@ki.se)

-----------------------------------------------------------

Reviewer #3: Alex McConnachie, Statistical Review

I thank the authors once again, for their patience in responding to my comments. I am happy with the answers given and the changes made to the paper.

Response: Thank you for the encouraging words. Please find below our point-to-point responses to your remaining comments. The page and line numbers refer to the clean version of the manuscript (.docx).

That said, the authors did not directly respond to my comment about not being able to find the distribution of the number of HPV tests per woman. I really think this should be made much clearer.

Apologies for not spotting this sooner, but on the latest reading, I figured out that the following is available in the flow diagram:

Study population (had at least one negative test): 337116 Had a subsequent positive test: 3263 Had at least one subsequent negative test: 57109 Had no subsequent tests: 276744

Response: We have now included the number of HPV tests in the text.

*“Within the study cohort, 3,263 women were tested positive for high-risk HPV during follow-up. 333,853 women were censored, among whom 57,109 had at least one negative test results during follow-up and 276,744 did not have a test (Figure 1).”* (Results section, page 11, lines 226-228)

In other words, 82% of the study population had a single negative test, any of whom could have acquired HPV after this point, but are assumed not to in the main analyses. It is notable that the interval censored results give confidence intervals of about the same width, despite the smaller sample size, since the number of events is the same. The weaker associations in the main analysis make sense, if women with a history of mental illness are less likely to attend for screening, and so tend to have longer follow-up after their last (or only) negative test, during which they are assumed to be event-free, thus diluting the overall association.

Response: Thanks for the good point. We have now further added this in the discussion.

*“Given that we have found women with mental illness are less likely to attend for the next screening, they tend to have longer follow-up after their last negative test, during which they are assumed to be infection-free, thus the infection rate among women with mental illness and the overall association might be underestimated. To address this, we conducted an interval-censored analysis on women with at least two HPV tests, restricting the follow-up as between the first and last tests, which showed similar but slightly stronger results. However, the right-censored analysis provided the opportunity to study the entire sample of the present study, instead of only women with at least two HPV tests during the study, and the differential adherence to HPV screening in relation to mental illness.”* (Discussion section, pages 14-15, lines 331-339)

Personally, I feel that the interval censored analysis, including 57109+3263=60372 women, is a more robust approach, but at least this is given in the supplements, and the overall conclusions of the main analysis appear correct, if a little biased.

Response: We have now moved the interval-censored analysis to the main body of the manuscript and revised the manuscript accordingly.

------------------------------------------------------------

Editorial comments.

We thank the authors for their extensive and comprehensive revision.

Response: We thank the editor for evaluating our work thoroughly. Please find below our point-to-point responses to your remaining comments. The page and line numbers refer to the clean version of the manuscript (.docx).

1. We agree with the view of the statistical reviewer (see above), and also find that interval censored analysis, including 57109+3263=60372 women, is a more robust approach. As such, we like to suggest that these results are moved the to main body of your manuscript and you revise your manuscript accordingly.
Response: We have now moved the interval-censored analysis to the main body of the manuscript and revised the manuscript accordingly.

2. For clarity, it may also be worth to include the HPV tests per woman in-text (with call-out to the flow diagram)
Response: We have now included the number of HPV tests in the text.

*“Within the study cohort, 3,263 women were tested positive for high-risk HPV during follow-up. 333,853 women were censored, among whom 57,109 had at least one negative test results during follow-up and 276,744 did not have a test (Figure 1).”* (Results section, page 11, lines 226-228)
